# Synthesis, antibacterial and antioxidant activities of Thiazole-based Schiff base derivatives: a combined experimental and computational study

**DOI:** 10.1186/s13065-021-00791-w

**Published:** 2021-12-23

**Authors:** Fitsum Lemilemu, Mamaru Bitew, Taye B. Demissie, Rajalakshmanan Eswaramoorthy, Milkyas Endale

**Affiliations:** 1 Department of Applied Chemistry, Adama Science and Technology University, P.O. Box 1888, Adama, Ethiopia; 2 Department of Chemistry, University of Botswana, Notwane Rd, P/bag UB 00704, Gaborone, Botswana; 3 Department of Biomaterials, Saveetha Dental College and Hospital, Saveetha University, Chennai, 600 077 India

**Keywords:** Schiff base, Thiazole, Antibacterial, Antioxidant, Molecular docking, Drug likeness, DFT analysis

## Abstract

**Background:**

Thiazole-based Schiff base compounds display significant pharmacological potential with an ability to modulate the activity of many enzymes involved in metabolism. They also demonstrated to have antibacterial, antifungal, anti-inflammatory, antioxidant, and antiproliferative activities. In this work, conventional and green approaches using ZnO nanoparticles as catalyst were used to synthesize thiazole-based Schiff base compounds.

**Results:**

Among the synthesized compounds, **11** showed good activities towards Gram-negative *E. coli* (14.40 ± 0.04), and Gram-positive *S. aureus* (15.00 ± 0.01 mm), respectively, at 200 μg/mL compared to amoxicillin (18.00 ± 0.01 mm and 17.00 ± 0.04). Compounds **7** and **9** displayed better DPPH radical scavenging potency with IC_50_ values of 3.6 and 3.65 μg/mL, respectively, compared to ascorbic acid (3.91 μg/mL). The binding affinity of the synthesized compounds against DNA gyrase B is within − 7.5 to − 6.0 kcal/mol, compared to amoxicillin (− 6.1 kcal/mol). The highest binding affinity was achieved for compounds **9** and **11** (− 6.9, and − 7.5 kcal/mol, respectively). Compounds **7** and **9** displayed the binding affinity values of − 5.3 to − 5.2 kcal/mol, respectively, against human peroxiredoxin 5. These values are higher than that of ascorbic acid (− 4.9 kcal/mol), in good agreement with the experimental findings. In silico cytotoxicity predictions showed that the synthesized compounds Lethal Dose (LD_50_) value are class three (50 ≤ LD_50_ ≤ 300), indicating that the compounds could be categorized under toxic class. Density functional theory calculations showed that the synthesized compounds have small band gap energies ranging from 1.795 to 2.242 eV, demonstrating that the compounds have good reactivities.

**Conclusions:**

The synthesized compounds showed moderate to high antibacterial and antioxidant activities. The in vitro antibacterial activity and molecular docking analysis showed that compound **11** is a promising antibacterial therapeutics agent against *E. coli,* whereas compounds **7** and **9** were found to be promising antioxidant agents. Moreover, the green synthesis approach using ZnO nanoparticles as catalyst was found to be a very efficient method to synthesize biologically active compounds compared to the conventional method.

**Supplementary Information:**

The online version contains supplementary material available at 10.1186/s13065-021-00791-w.

## Introduction

The design and synthesis of organic compounds for medicinal applications is an important area of medicinal chemistry. When the organic compounds are complexed with transition metals, there has been observed an increase in the potency of therapeutic compounds [1–5]. Medicinal inorganic chemistry is also becoming the most attractive and growing research area with great scope to explore the potential of Schiff base compounds and their metal complexes for therapeutic applications [3, 6]. In this aspect, many Schiff base compounds have been synthesized since 1864 [7], with wide areas of biological applications [7–11]. They are important building blocks for new materials with promising electronic, mechanical or biological properties [12]. They have attracted a great deal of interest in recent times for the design of new biologically significant compounds. More than 60% of all the known organic compounds are heterocyclic compounds [13, 14]. However, the range of easily accessible and suitable functionalized heterocyclic building blocks for the synthesis of structurally diverse libraries of therapeutics is limited. Compounds containing azomethine group (–C=N–), Schiff bases (SBs), are usually synthesized by condensation of primary amines and active carbonyl groups [15]. Such compounds display promising pharmacological properties as anti-bacterial [16], antifungal [17], anti-cancer [18, 19], antioxidant [19, 20], antimalarial [21] anti-inflammatory [22], antiviral [23] and anti-proliferative properties [24]. They have been utilized as synthons to prepare biologically and industrially active compounds via ring closure, cycloaddition, and replacement reactions [15, 25]. The pharmacophore potential of SBs is because of their ability to form complex compounds with metal ions in the active center of many enzymes involved in metabolism [16]. Moreover, the nitrogen atom of imine (–C=N–) is suggested to form a hydrogen bond with the active centers of cell constituents and thereby interfering the normal cell processes [2, 9]. Thiazole moiety is one of the important pharmacophores in drug discovery and development with a wide range of therapeutic targets including antibacterial [26], anti-diabetic [18, 19], anti-cancer [27], anti-inflammatory [22] and antiviral [23, 28]. Although the exact biochemical mechanism of the antioxidant and antibacterial activities of thiazole-based Schiff base compounds are not well explored, the likely mechanism for their antioxidant activities have been suggested to be due to their ability to donate hydrogen to free radicals, ability to bind to reversible oxygen redox system from biochemical reactions, and their potential to deactivate many cellular enzymes. More importantly, their ability to inhibit aminoacyl-tRNA synthesis pathways is suggested as the possible mechanism to act as antibacterial agents [10, 11].

In the present work, we report five thiazole-based Schiff bases designed and synthesized using conventional and green synthesis methods. The absorption, distribution, metabolism, excretion, and toxicity (ADMET) profile predictions, DFT calculations, molecular docking study against DNA gyrase B and human peroxiredoxin 5, antibacterial and antioxidant activities of the five thiazole-based Schiff base derivatives are also presented.

## Experimental

**Scheme 1:**
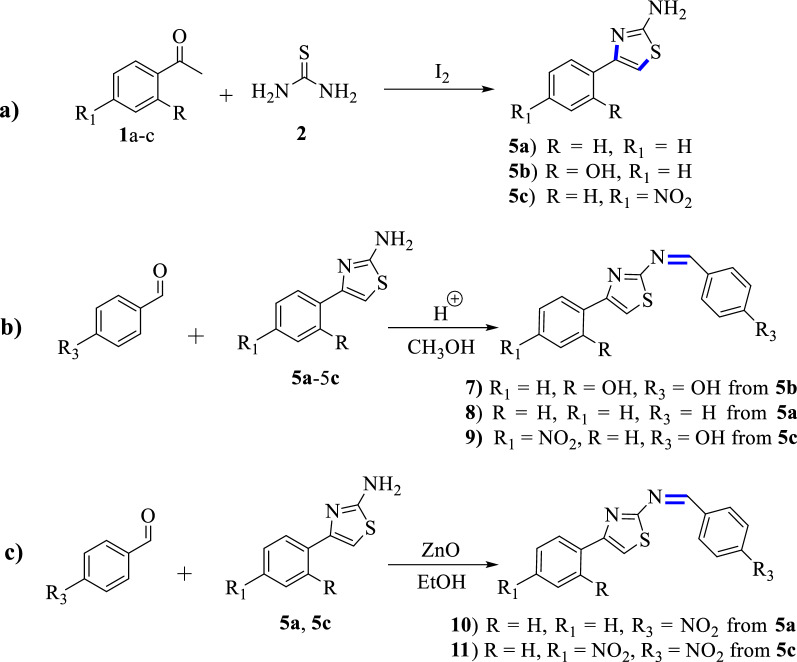
General schemes for the synthesis of Schiff base-based thiazole and its derivatives: **a** synthetic steps for the intermediate products, **b** conventional synthetic steps, and **c** green synthetic steps. Note that **7**, **8** and **9** were synthesized from **5b**, **5a** and **5c**, respectively, whereas **10** and **11** were synthesized from **5a** and **5c,** respectively

Five thiazole-based Schiff bases were designed by altering the substituents on the phenyl ring of acetophenone and benzaldehyde (Scheme 1). Benzaldehyde (99% AR), 4-nitrobenzaldehyde (99% AR), 4-hydroxybenzaldehyde (98.8), acetophenone (AR), 4-nitroacetophenone (98%), 2-hydroxy acetophenone (95%), thiourea (99% AR), iodine beads, ethanol (absolute 99.8%), glacial acetic acid (99.5%), methanol (98.8% AR), ethyl acetate (99.5% AR), diethyl ether, sodium thiosulphate (99.5%), sodium carbonate anhydrous (99.5%), DPPH (Sigma-Aldrich) and others were purchased from Loba Chemie PVT Ltd, Addis Ababa (Neway PLC), Ethiopia. The synthesized compounds were purified by recrystallization and silica gel column chromatography (using silica gel 60–120 mesh size). Thin Layer Chromatography (TLC) profile was monitored using thin layer chromatography plate (Merck Silica gel 60 F254) and visualized using UV–Vis lamp at 254 and 365 nm. Melting points were determined using open capillary tubes (Thiele tube) filled in oil, where the melting points were measured in a range of values. NMR (^1^H and ^13^C) spectra were recorded using Bruker Avance 400 MHz spectrometer using deuterated chloroform (CDCl_3_) and dimethyl sulfoxide (DMSO-*d*_6_) solvents. Absorption spectra were recorded using double beam UV–Visible spectrophotometer (model 2201, India). FTIR spectra were recorded in KBr from 4000 to 400 cm^−1^ using Fourier-Transform Infrared Spectrometer (FTIR, Shimadzu Corporation, Japan).

### Synthesis procedures for compounds 5a–c

Thiourea (40 mmol, 2 molar equiv.) was mixed with iodine beads (20 mmol, 1 molar equiv.) in a mortar and the mixture was homogenized with a pestle [29]. The solids were scratched off into a 250 mL round-bottom flask with a magnetic stirring bar and mixed with the corresponding acetophenone, 2-hydroxy acetophenone and 4-nitro acetophenone (20 mmol, 1 molar equiv.) (Scheme 1, Path **a**) [30, 31]. The mixture was refluxed and stirred for 3 h at 100 °C in an oil bath under air-cooled condenser. Aqueous solution of sodium thiosulfate pentahydrate (5%) was added (with concomitant mechanical disruption of the solid matrix) in sufficient amount to reduce the remaining iodine (until brown colour disappeared) [29, 31]. The suspension was filtered off and carefully washed with ~20 mL of diethyl ether (caution: product partially soluble) to remove the residues of unreacted acetophenone and its derivatives [29, 31]. The crude product was dissolved in hot water, filtered, and the hot filtrate was adjusted to pH = 9 using aqueous Na_2_CO_3_ anhydrous [31]. The products were collected by suction filtration. The physical properties are presented in Additional file 1: Table S1.

### Synthesis of thiazole-based Schiff bases (7–9)

A solution of phenyl thiazole (25 mmol) and benzaldehyde and its derivatives (25 mmol) were mixed in methanol (40 mL) (Scheme 1, Path **b**) [32]. The reaction mixture was refluxed for 5 h at 65 °C. The mixture was left to stand at room temperature overnight and concentrated using rotary evaporator. The residue was washed with *n*-hexane and filtered. Then it was hydrolyzed in water and extracted with EtOAc. Powders of the compounds (**7**–**9**) were obtained after drying with Na_2_SO_4_ [33]. The physical properties of the synthesized compounds are presented in Additional file 1: Table S1.

### Green synthesis of Schiff bases (10 and 11)

A solution of acetophenonethiazole (0.4 g, 2.3 mmol) and *p*-nitrobenzaldehyde (0.34 g, 2.3 mmol), and *p*-nitroacetophenone thiazole (0.3 g, 13.5 mmol) and *p*-nitrobenzaldehyde (0.205 g, 13.5 mmol) (Scheme 1, Path **c**) [34] were mixed in absolute ethanol (30 mL) and refluxed for 4 h in the presence of 15% ZnO nanoparticles load at 78 °C for compounds **10** and **11**, respectively. The mixture was poured into 150 mL of crushed ice, while stirring with glass road and powders obtained were washed with cold ethanol and diethyl ether to remove unreacted amine, aldehyde and its derivatives [33] (Scheme 1**)**. The physical properties of the synthesized compounds are presented in Additional file 1: Table S1.

### Antibacterial activity

In vitro antibacterial activities of the test compounds against multi drug resistant bacterial strains were studied by disk diffusion assay [35]. The media was prepared by dissolving 38 g of Mueller Hinton agar (MHA) medium in 1000 mL of distilled water and autoclaved at 121 °C for 15 min. The autoclaved medium was poured into sterile plates (20–25 mL/plate) and allowed to solidify under sterile condition at room temperature.

The bacterial cultures were inoculated into the nutrient broth (inoculation medium) and incubated overnight at 37 °C [36, 37]. Inoculated medium containing a 24 h grown culture was added aseptically to the nutrient medium and mixed systematically to get an even distribution. The solution was poured into ~20 mL of sterile MHA in sterile culture plates and allowed to attain room temperature [36, 37]. Sterile agar-disc diffusion previously soaked in a known concentration (100 μg/mL and 200 μg/mL per disc) of the synthesized compound and standard drugs were prepared in DMSO using nutrient agar tubes and carefully placed at the center of the labeled seeded plate [36, 37]. Mueller–Hinton sterile agar plates were seeded with indicator bacterial strains (1.3 × 10^8^ cfu/mL) and allowed to stay at 37 °C for 3 h. Sterile filter paper disks with a diameter of 6 mm were placed over these plates. Finally, the plates were incubated at 37 °C for 24 h [35–37].

The mean inhibition zones were measured with a ruler and compared with the positive control (a disk containing amoxicillin) of the same concentration in millimeter. DMSO was used as a negative control during the whole test. The mean inhibition zone (MIZ) was expressed as mean value ± standard deviation.

### Bacterial culture

The bacterial species, isolated from stored stool specimens and identified according to biochemical tests, were obtained from Adama Public Health Research & Referral Laboratory Center, Ethiopia. It is important to note that no patient was involved during specimen collection. The species were then cultured overnight using Eosin Methylene Blue (EMB) agar [37]. Gram-negative strains (*Escherichia coli* (ATCC25922) and *Pseudomonas aeruginosa* (ATCC27853)) and Gram-positive strains (*Staphylococcus aureus* (ATCC25923) and *Streptococcus pyogenes* (ATCC19615)) were used to test the activities of the synthesized compounds. Different concentrations were prepared from the synthesized compounds by dissolving 5 mg of each compound in 5 mL DMSO to make 1 mg/mL of standard solution. The experiment was performed in triplicates. The standard solution was serially diluted to furnish 100 μg/mL and 200 μg/mL samples for each synthesized compound. The concentrations of each sample were incorporated into sterile blank paper discs and dried at 37 °C [38].

### Antioxidant activity

In vitro antioxidant activities of the synthesized compounds were assessed according to a reported method [39]. In brief, 0.01 mg/mL (0.001% (w/v)) solution of DPPH in methanol and DMSO was prepared, 1 mL of this solution was poured into 4 mL of the synthesized samples in methanol to furnish four different concentrations (1.25, 2.5, 5, 10 μg/mL) [40]. The control was made by adding 1 mL of DPPH solution to 4 mL of methanol or 4 mL of DMSO. Absorbance was measured against blank at λ_max_ of 517 nm using UV–Visible Spectrophotometer (SM-1600 Spectrophotometer, India) [41]. Ascorbic acid was used as a positive control. The activity was expressed in IC_50_ in which the concentration of the compounds is required to give a 50% decrease in absorbance compared to that of the control solution. The percentage inhibition of the synthesized compounds against DPPH was calculated using [42]:

$$\% {\text{ Inhibition }} = \, \left[ {\left( {{\text{A}}_{{{\text{control}}}} - {\text{ A}}_{{{\text{sample}}}} } \right)/{\text{A}}_{{{\text{control}}}} } \right] \times {1}00,$$

where A_control_ is the absorbance of the control, A_sample_ is the absorbance of the test compounds.

### Pharmacokinetic studies

Physicochemical properties, drug likeness and pharmacokinetic properties (ADME) of the designed thiazole-based Schiff base compounds were determined using the SwissADME Web server [43]. The percent absorption (%Abs) of the synthesized compounds was calculated using the formula %Abs = 109–0.345 TPSA [44]. The toxicity profile of the synthesized compounds was predicted using ProTox-II Web tool [45].

### Density functional theory study

Geometry optimizations and frequency calculations of the synthesized compounds (**7–11**) were performed using the Gaussian 16 program package [46] and the results were visualized using GaussView 06 software. Density functional theory (DFT) and time dependent density functional theory (TD-DFT) calculations were performed using the B3LYP hybrid functional [47–49] together with 6–311++G(d,p) basis set [49]. Grimme’s dispersion correction [50] was employed to treat non-bonding interactions during the calculations. Such a combination of functional and basis sets has been used in previous studies [51, 52]. Solvent effects were corrected using the polarizable continuum model in its integral equation formalism (IEFPCM) [53] together with either methanol or DMSO solvents to mimic the experimental conditions. The transition states were calculated using the quadratic synchronous transit (QST3) method, which requires not only an initial guess for the transition-state geometry, but also the optimized structures of the reactants and products. The optimized transition state structures were confirmed by the presence of an appropriate single imaginary vibrational frequency. The changes in Gibbs free energies were all calculated at 298.15 K and 1 atm.

The optimized geometries were confirmed to be real minima without any imaginary vibrational frequency by performing vibrational frequency calculations. The frontier molecular orbitals (highest occupied molecular orbital, HOMO, and lowest unoccupied molecular orbital, LUMO), energy gap (Δ*E* = *E*_LUMO_ − *E*_HOMO_), electronegativity (*χ* = − ½ (*E*_HOMO_ + *E*_LUMO_)), electronic chemical potential (*μ* = ½ (*E*_HOMO_ + *E*_LUMO_) = − *χ*), global chemical hardness (*η* = ½ (_*E*LUMO_ − *E*_HOMO_)), global softness (σ = 1/2η), global electrophilicity index (*ω* = *μ*^*2*^*/2η*), and nucleophilicity index (*Nu* = 1/*ω*)*,* dipole moment, natural atomic charges (NAC) and molecular electrostatic potential (MEP) of the synthesized compounds were also calculated and analyzed at the same level of theory [54]. The polarizability unites were converted from au into angstrom (au/1.88973), and then to Bohr^3^ (angstrom * 0.529177) using standard conversion factors.

### Molecular docking

Molecular docking studies of the synthesized compounds (**7**–**11**) against target proteins (human peroxiredoxin 5 PDB ID: 1HD2 and *E. coli* DNA gyrase B PDB ID: 6F86) were performed using AutoDock Vina 4.2 (MGL tools 1.5.7) following standard protocol [55, 56]. The three- and two-dimensional construction of the compounds, energy minimization of the synthesized compounds, docking simulations, crystal structures of the receptor molecules (*E. coli* DNA gyrase B, PDB ID: 6F86 [57]; and human peroxiredoxin 5, PDB ID: 1HD2 [58], protein preparation, docking algorithm and ligand representations were conducted following the same procedure and protocols reported in our previous work [59, 60].

## Results and discussion

### Synthesis and reaction mechanisms

**Fig. 1:**
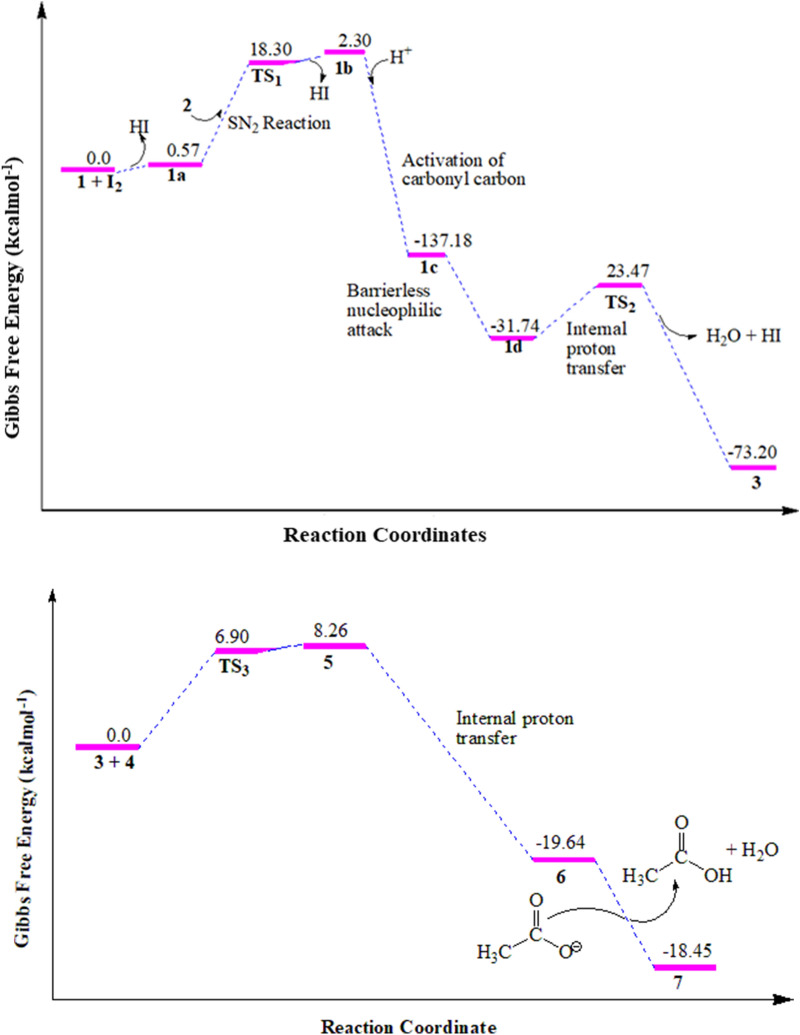
Free energy profile for the conventional synthesis of compounds **3** (top) and **7** (bottom). Note that the change in energies were calculated with respect to the reaction paths.

Five novel compounds were synthesized and characterized using spectroscopic methods with yields from 68.3 to 83.3%. Shorter reaction time was achieved for ZnO nanoparticle catalyzed approach compared to the conventional glacial acetic acid catalyzed reactions under reflux conditions. Physical properties and NMR spectral data of the synthesized compounds are presented in supporting information (Additional file 1: Figs. S1–S10). The change in Gibbs free energies of the reaction mechanisms for the synthesis of compounds **3** and **7** using the conventional method was analyzed from DFT calculations. The results are presented in Fig. 1.

Free energy profile of the intermediates and the transitions states involved in the reaction mechanisms are presented in Fig. 1. The S_N_2 reaction of **1a** with **2** in a neat condition resulted in the formation of the first transition state (TS_1_) with 18.3 kcal/mol energy barrier. The removal of HI from TS_1_ gives unstable intermediate **1b** (2.3 kcal/mol). The HI is further used to activate the carbonyl carbon, which yields a very stable intermediate **1c** (− 137.18 kcal/mol). Cyclization of compound **1c** through a barrierless nucleophilic attack yields the formation of **1d.** Internal proton transfer from the imine to hydroxyl group followed by the abstraction of a proton by I^**−**^ takes the reaction to the formation of the second transition state (TS_2_) with an energy barrier of 23.47 kcal/mol**.** The concomitant removal of water and hydrogen molecules from TS_2_ yields the formation of compound **3** with a change in Gibbs free energy of − 73.20 kcal/mol (Fig. 1), assuring the formation of a stable reaction intermediate.

**Fig. 2:**
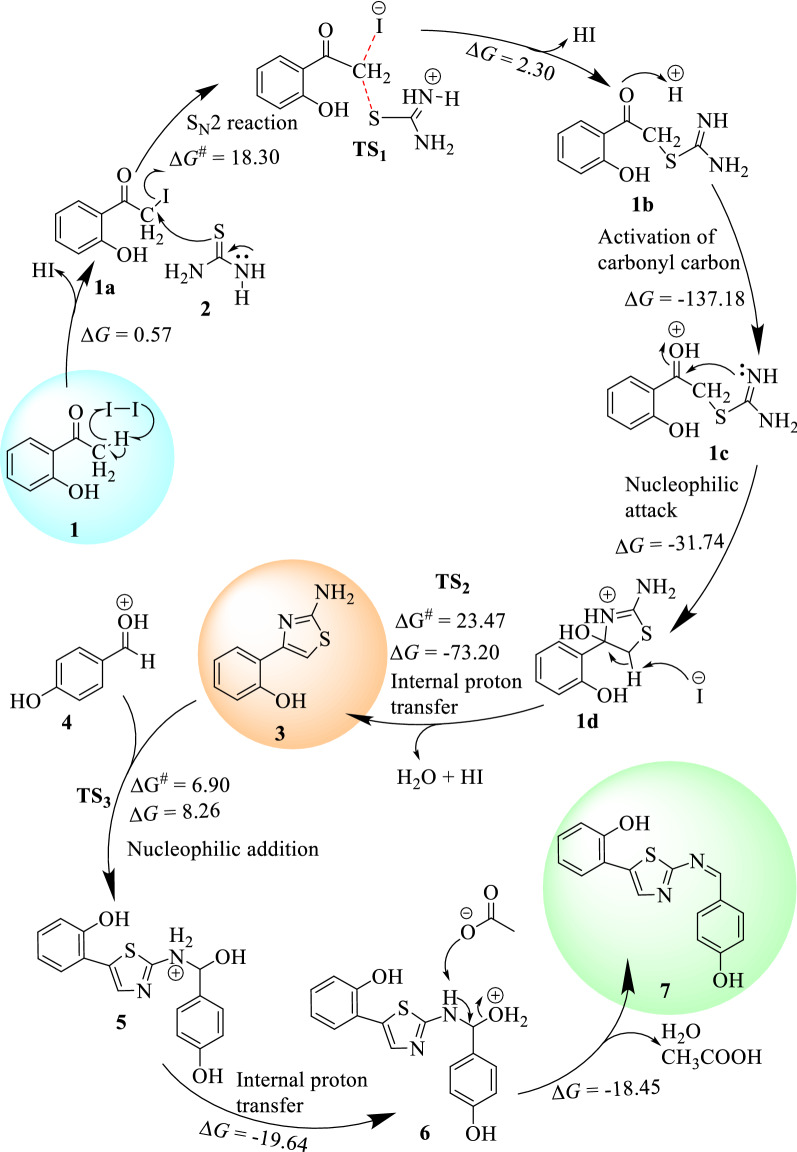
The plausible reaction mechanism of compounds **3** and **7** calculated using B3LYP-GD3/6-311+ +G(d,p)/Methanol. At the start of the reaction, it proceeds via an S_N_2 reaction mechanism followed by the formation of first transition state (TS_1_). The second (TS_2_) and third (TS_3_) transition states follow nucleophilic reactions

The nucleophilic addition of compound **3** and **4** in the presence of acetic acid gives the formation of the third transition state (TS_3_) with activation energy barrier of 6.9 kcal/mol which gives the formation of compound **5** (1.36 kcal/mol). The internal proton transfer from the amine of imine was suggested to form compound **6** (− 19.64 kcal/mol) which yields the target compound **7** (− 18.45 kcal/mol) via deprotonation and dehydration (Fig. 1). Overall, the formation of compound **7** follows a radical reaction to generate α-halo carbonyl compounds, nucleophilic substitution, and nucleophilic addition reaction mechanisms. The summarized plausible reaction mechanism, based on the change in Gibbs free energies of the reaction steps, for the synthesis of **3** and **7** is presented in Fig. 2.

**Fig. 3:**
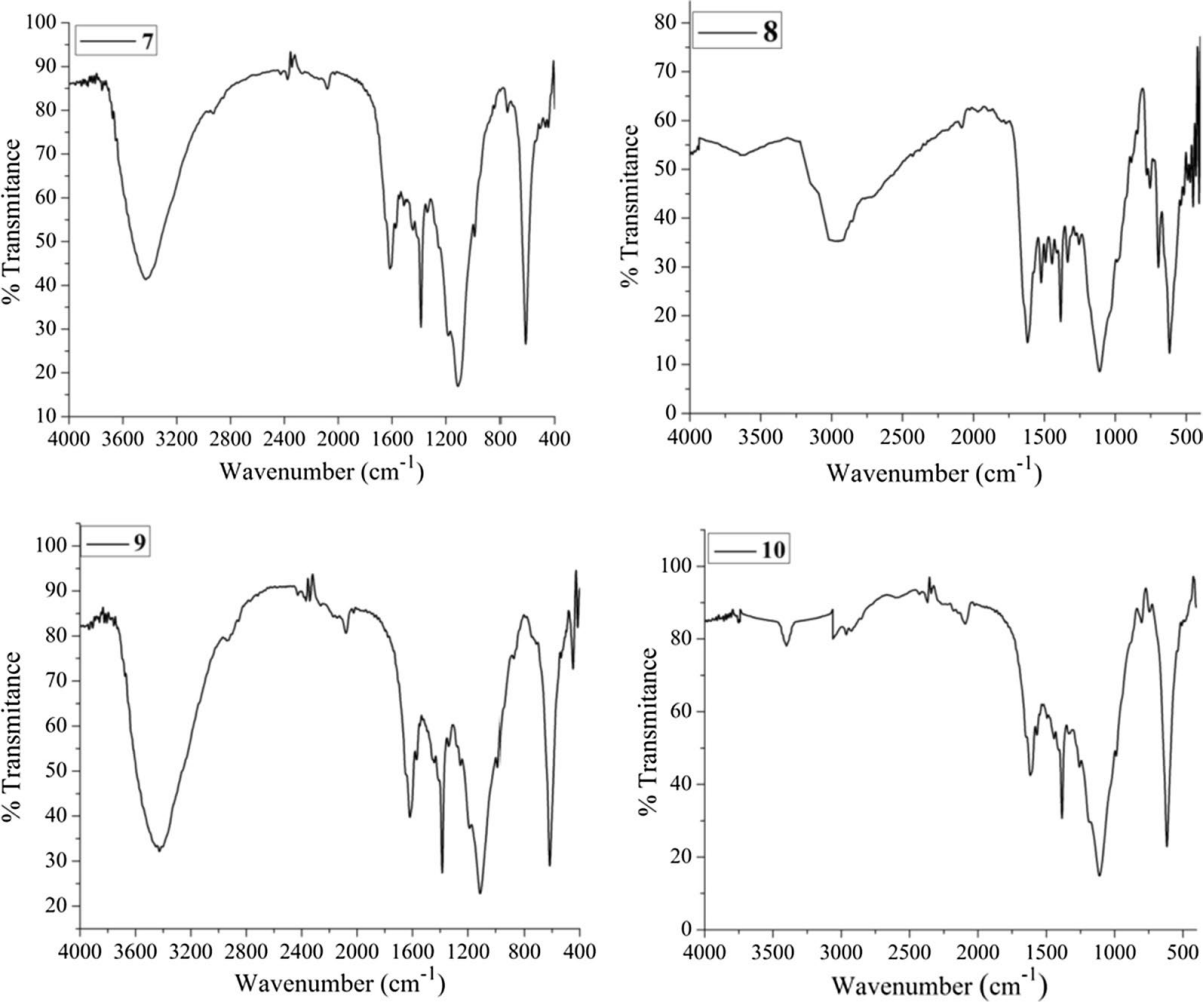
FTIR spectra of the synthesized compound **7**–**10**

The experimental FTIR spectra of compounds **7**–**10** are presented in Fig. 3, whereas that of **11** is presented in Additional file 1: Fig. S10. To further support the synthesis of the compounds, we compared the experimental and DFT calculated IR vibrational spectra of the five compounds. The results are in good agreement with each other (Table 1). The absence of amine peaks and the appearance of a peak (experimental/theoretical) at 1616/1618 cm^−1^, 1632/1661 cm^−1^, 1632/1582 cm^−1^, and 1608/1657 cm^−1^ indicates the characteristic peaks for C=N stretching of the imine groups of compounds **7–10** (Fig. 3; Table 1).

**Table 1:** The FTIR spectral data (experimental/computational) data of the synthesized compounds

Compound	Functional group assignments					
	ν(O–H)	ν (C–H)	ν (C=N)	ν (Thaizole nucleus)	ν (C–S)	ν (N–O)
7	3440/3706	2928/3095	1616/1618	1376 and 1104/1581 and 1152	608/654	–
8	–	2928/3110	1632/1661	1376 and 1096/1326 and 1184	632/663	–
9	3440/3704	2952/3044	1632/1582	1388 and 1104/1294 and 1108	620/648	1388/1478
10	–	2952/3106	1608/1657	1376 and 1115/1325 and 1176	608/669	1376/1479
11	–	2936/3046	1611/1611	1384 and 1104/1305 and 1089	608/686	1384/1488

Absorption frequencies from DFT were scaled by 0.975 scaling factor

**Fig. 4:**
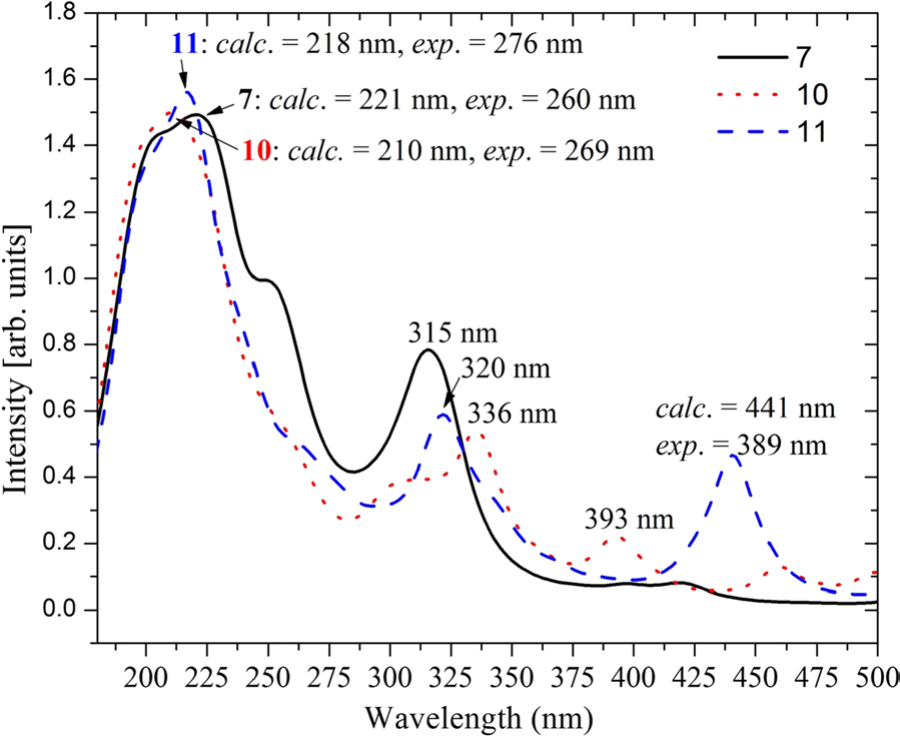
Comparison of the experimental absorption wavelengths with the corresponding B3LYP-GD3/6-311++G(d,p)/PCM/DMSO calculated results. The calculated spectra were red-shifted by 30 nm for better comparison with the experimental results

UV–Vis absorption spectra of the synthesized compounds were recorded from 200 to 800 nm at room temperature using methanol (for **8** and **9**), and DMSO (for **7**, **10** and **11**) (Fig. 4). The computational/experimental maximum absorption bands (Fig. 4) for the synthesized compounds appeared at 221/260 nm, 203/214 nm, 215/260 nm, 210/269 nm, and 218/276 nm for compounds **7–11**, respectively. These λ_max_ absorption bands are attributed to the transition of electrons from π to π* orbitals as the HOMO–LUMO systems are mainly localized over the π-system of the imine part of the molecule (vide infra), in agreement with previous reports [61] (Additional file 1: Fig. S11). Even though satisfactory results are obtained from the DFT calculations, there are differences between the experimental and calculated maximum absorption wavelengths for some of the compounds. This can be attributed to missing treatment of molecular packing forces in the computational method.

### Antibacterial activity

**Fig. 5:**
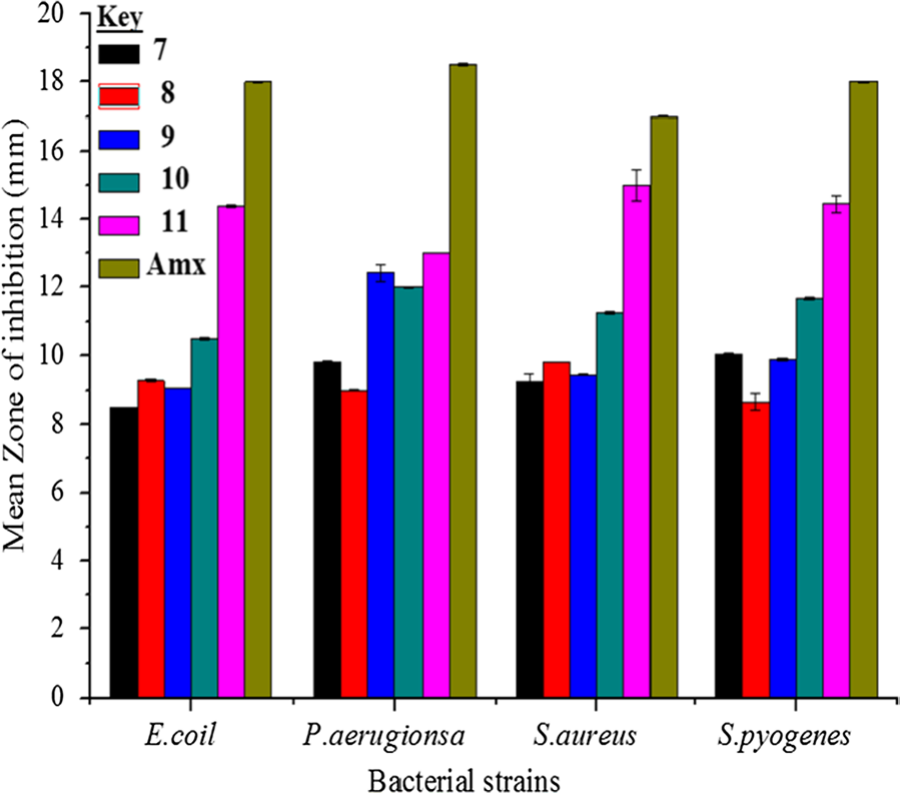
Mean inhibition zone of the synthesized compounds in mm (mean ± SD) at 200 µg/mL. The inset stands for the type of compounds

Thiazole containing compounds were proved to have potent antimicrobial activity against multidrug-resistant strains of *S. aureus* [62]. In this work, in vitro antibacterial activities of the synthesized compounds were done against four clinical bacterial isolates (Fig. 5). The synthesized compounds were less active against Gram-negative rather than Gram-positive bacteria. Compounds **10** and **11** displayed good activities against *E. coli* with MIZ of 10.50 ± 0.02 and 14.40 ± 0.04 mm diameter, respectively, compared with amoxicillin (18.00 ± 0.01 mm) at 200 μg/mL (Table 2). Compounds **8** and **11** showed good activities against *S. aureus*, while compounds **7**, **9** and **10** showed good activities against *S. pyogenes* (Table 2).

**Table 2:** Mean inhibition zone of the synthesized compounds in mm (mean ± SD) at different concentrations and their comparison with amoxicillin

Compounds	Conc. in μg/mL	Zone of inhibition (mm) mean ± SD			
		*E. coli*	*P. aeruginosa*	*S. aureus*	*S. pyogenes*
**7**	100	7.31 ± 0.03	8.00 ± 0.02	8.60 ± 0.02	7.95 ± 0.25
**8**		7.45 ± 0.02	7.95 ± 0.30	7.20 ± 0.04	7.25 ± 0.01
**9**		9.00 ± 0.01	10.00 ± 0.01	8.00 ± 0.04	8.67 ± 0.30
**10**		8.00 ± 0.02	7.00 ± 0.01	7.00 ± 0.00	7.95 ± 0.25
**11**		12.00 ± 0.01	12.43 ± 0.02	11.00 ± 0.00	13.00 ± 0.01
**7**	200	8.50 ± 0.01	9.80 ± 0.25	9.23 ± 0.02	10.03 ± 0.04
**8**		9.25 ± 0.04	9.00 ± 0.01	9.80 ± 0.45	8.67 ± 0.25
**9**		9.08 ± 0.01	9.43 ± 0.23	9.56 ± 0.25	9.89 ± 0.05
**10**		10.50 ± 0.02	12.00 ± 0.01	11.25 ± 0.15	11.67 ± 0.30
**11**		14.40 ± 0.04	13.00 ± 0.02	15.00 ± 0.01	14.45 ± 0.25
Amoxicillin	100	16.00 ± 0.00	16.00 ± 0.45	15.00 ± 0.01	16.00 ± 0.55
	200	18.00 ± 0.01	8.50 ± 0.45	17.00 ± 0.04	18.00 ± 0.02

Compound **11** showed strong antibacterial activity against Gram-negative (*P. aeruginosa*) and Gram-positive (*S. aureus*) with MIZ of 13.00 ± 0.02 and 15.00 ± 0.01 mm compared with amoxicillin (18.50 ± 0.45 and 17.00 ± 0.04 mm), respectively, at 200 μg/mL (Table 2; Fig. 5). This suggests that compound **11** is potentially a promising therapeutic antibacterial agent. The good activity exhibited by compound **11** compared to others (**7**–**10**) is due to the appearance of electron withdrawing groups (–NO_2_) in the structure of the compound that would create localized electron deficient sites which can facilitate a feasible condition for interaction with biomolecules (proteins, amino acids, nucleic acids, and enzymes) [56–58]. The high antibacterial activity of compound **11** can also be attributed to its high dipole moment (vide infra) [63].

### Antioxidant activity

Oxidative stress is a cause of cancer as well as neurological and cardiovascular diseases which are the leading causes of death [41]. Compounds **7–11** were evaluated for their antioxidant activities using DPPH assay. Compounds** 7** (80.89%, IC_50_ value 3.6) and **9** (70%, IC_50_ value 3.65 μg/mL) (Table 3; Additional file 1: Figs. S12, S13) showed promising antioxidant potential at concentration of 10 μg/mL compared to ascorbic acid (91.2%, IC_50_ value 3.98 μg/mL). The high antioxidant activity of compound **7** could be due to electron donating –OH substituents [64, 65]. The quantum mechanical descriptors of the compounds support these experimental results (vide infra).

**Table 3:** DPPH scavenging activity of the synthesized compounds

Concentration (μg/mL)	Ascorbic acid	**7**	**8**	**9**	**10**	**11**
1.25	22.69%	15.73%	17.13%	17.13%	21.60%	15.40%
2.50	35.00%	58.15%	42.70%	64.50%	27.20%	22.00%
5.00	73.10%	71.35%	43.80%	66.00%	59.83%	58.15%
10.00	91.20%	80.89%	75.80%	70.00%	74.50%	88.80%
IC_50_	3.98	3.60	5.56	3.65	5.36	5.14

### In silico pharmacokinetics (ADME) and drug likeness studies analysis

To shed more light on the in vitro analysis of the antibacterial and antioxidant activities of the synthesized compounds, in silico pharmacokinetics and drug-likeness studies were conducted. The results are presented in Additional file 1: Tables S2, S3. The molecular weight of the molecules ≤500), the number of hydrogen bond acceptors (≤10), number of hydrogen bond donors (≤5) and the lipophilicity (iLogP) values ≤5) were used to follow Lipinski’s rule of five for drug like molecular nature of a molecule [66]. The SwissADME predicted results showed that the synthesized compounds (**7**–**11**) satisfy Lipinski’s rule of five [43] with zero violations for their drug like molecular nature (Table 4).

**Table 4:** In silico drug-likeness predictions of synthesized compounds computed by SwissADME

Compounds	HBAs^a^	HBDs^b^	MR	TPSA	iLogP	Lipinski^b^ violations
**7**	4.00	2.00	85.29	93.95	2.54	–
**8**	2.00	0.00	81.25	53.49	3.07	–
**9**	5.00	1.00	92.09	119.54	2.10	–
**10**	4.00	0.00	90.07	99.31	2.71	–
**11**	6.00	0.00	98.89	145.13	2.34	–
Amoxicillin	6.00	4.00	94.59	158.26	0.95	–

^a^HBAs = number of hydrogen bond acceptors

^b^HBDs = number of hydrogen bond donors, MR = molar refractivity, and TPSA = topological polar surface area. Molecular weights for all the compounds are less than 400 g/mol

The SwissADME predicted LogP values are in the range from 2.10 to 3.07 assuring optimal lipophilicity of the synthesized compounds. In addition, TPSA of the synthesized molecules were used to predict percent absorption descriptors. The predicted TPSA value (in Å^2^) are in the range from 39.99 to 145.13 inferring very good intestinal absorption of compounds **7**–**10,** and relatively poor intestinal absorption for compound **11**. It has been suggested that molecules with a TPSA of 140 Å^2^ and above would be poorly absorbed (< 10% fractional absorption), while compounds with a TPSA 60 Å^2^ would be well absorbed (> 90%) [67]. High degree of absorption was predicted for compounds **7**–**10**. The predicted human skin permeability coefficients (logKp) values of the synthesized compounds for their human stratum corneum penetration were found to be in the range of − 4.37 to − 5.59 cm/s, inferring low skin permeability [43]. A relatively less skin permeant was predicted for compound **7**.

Pharmacokinetically, high GI absorption for **7**, **8** and **9**, BBB permeate for **8** were predicted. None of the synthesized compounds were predicted as Pg-p substrate (Additional file 1: Table S2). SwissADME predicted results also suggested that compound **9** is a better active therapeutic agent compared to other synthesized compounds. Besides, the interaction of therapeutic molecules with cytochromes P450 (CYP) isoforms as substrate of these enzymes has been proposed to screen a therapeutically active molecule. It is reported that the inhibition of CYP isoenzymes is certainly one major cause of pharmacokinetics-related drug-drug interactions [68, 69], thereby leading to toxic/adverse effects due to the lower clearance and accumulation of the drug or its metabolites [70]. In this work, except to CYP1A2 isoform, most of the synthesized compounds were substrates of (CYP) isoforms. The results further suggest that the synthesized compounds have good drug-like molecular nature.

Absence of toxicity data is one of the life threatening factors for choosing a compound as a therapeutic candidate [71]. Herein, the organ toxicity (hepatotoxicity) and toxicological endpoints (carcinogenicity, immunotoxicity, mutagenicity and cytotoxicity) of the five compounds were predicted. The ProTox-II predicted organ toxicity results showed that all the synthesized compounds show hepatotoxicity and mutagenicity toxicological endpoint. Besides, all the compounds show no toxicity towards immunotoxicity toxicological endpoint. Carcinogenicity toxicological endpoints were predicted for compounds **7**, **9**, **10** and **11** (Additional file 1: Table S3). The results showed that LD_50_ values of the synthesized compounds are class three (50 ≤ LD_50_ ≤ 300), indicating that they are toxic [45].

### Quantum mechanical descriptors

It is known that high eigenvalue of HOMO and small band gap energy between HOMO and LUMO of a compound are associated with its high antioxidant activity [72]. Having this in mind, we analyzed the quantum mechanical descriptors of all the compounds. The results are presented in Table 5. The HOMO and LUMO frontier molecular orbitals were used to analyze the relative reactivity and describe the wave function distribution of the molecules. The energy gaps (in **eV**) were calculated to be 2.242, 1.795, 2.095, 1.805 and 2.181 for compounds **7–11**, respectively (Table 5). The results indicated that the synthesized compounds have good reactivity. The results further revealed that compound **8** has the minimum HOMO–LUMO energy gap signifying that it has high chemical reactivity and sizable intramolecular charge transfer.

**Table 5:** Quantum chemical descriptors of the synthesized compounds in (eV)

Cpds	*E*_HOMO_	*E*_LUMO_	*E*_g_	***χ***	µ	η	σ	ω	Nu	Polarizability (Bohr^3^)	Dipole moment (Debye)
**7**	− 5.880	− 3.638	2.242	4.759	− 4.759	1.121	0.446	10.10	0.099	97.72	6.083
**8**	− 5.972	− 4.177	1.795	5.074	− 5.075	0.898	0.557	14.35	0.069	108.50	6.249
**9**	− 6.242	− 4.147	2.095	5.194	− 5.195	1.048	0.477	12.88	0.077	112.88	14.629
**10**	− v6.091	− 4.286	1.805	5.188	− 5.189	0.903	0.554	14.91	0.067	118.58	5.497
**11**	− 6.433	− 4.252	2.181	5.342	− 5.343	1.091	0.459	13.09	0.076	117.12	10.164

Compounds **9–11** showed large electronegativity values 5.194 eV, 5.188 eV and 5.3425 eV, respectively. The structural differences brought the observed electronegativity differences between compounds **10** (single –NO_2_ substituent) and **11** (double –NO_2_ substituent) (Table 5). The presence of electron donating hydroxyl substituent in compound **7** reduced its electronegativity (4.759 eV) relative to its structurally analogous compound **8** (5.074 eV). On the other hand, relatively large dipole moment values were calculated for compound **9** (14.629 Debye) and compound **11** (10.164 Debye) inferring potentially good antimicrobial activity of the synthesized compounds (Additional file 1: Fig. S14).

**Fig. 6:**
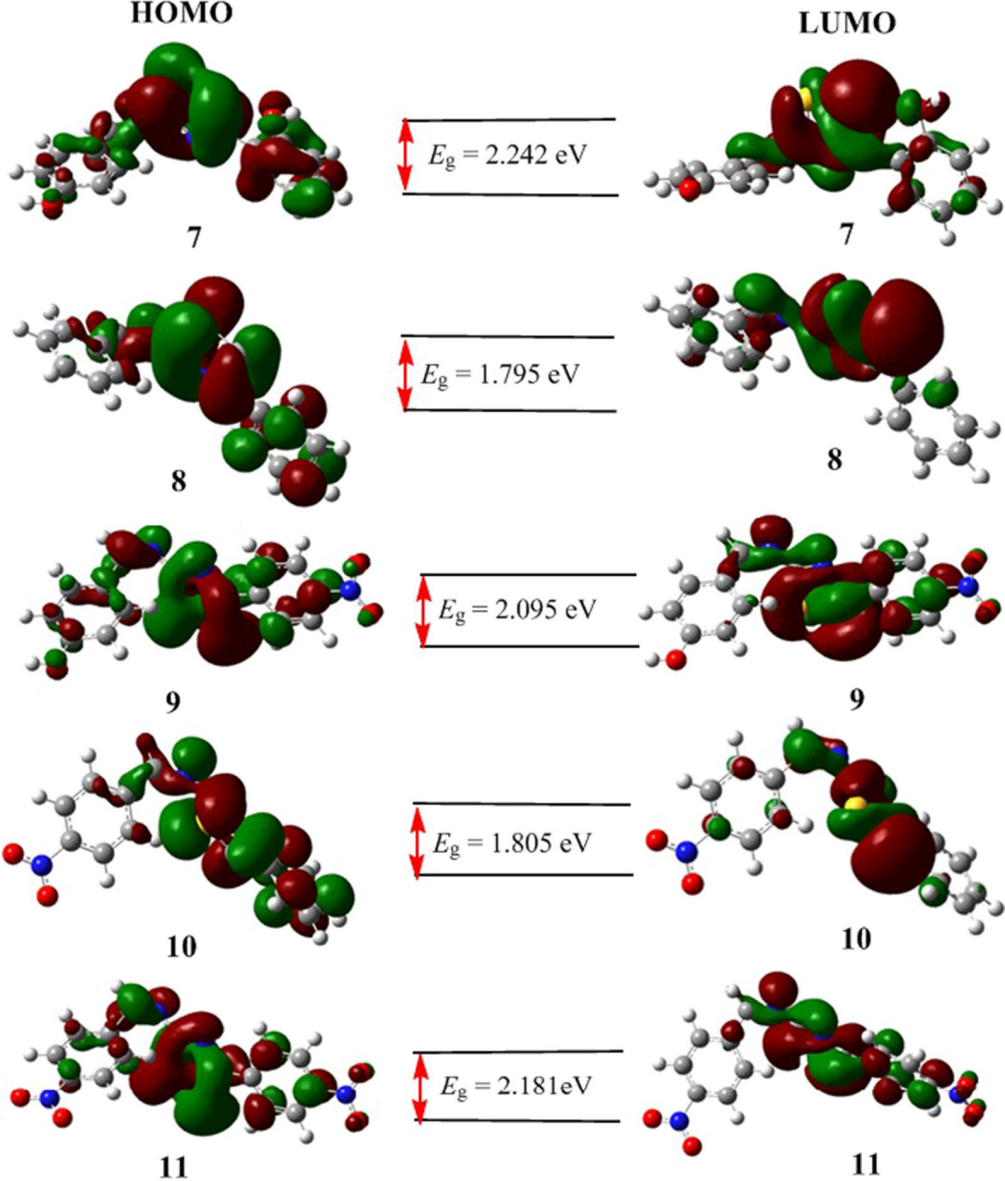
HOMO–LUMO distributions of the synthesized compounds (**7**–**11**). *E*_*g*_ represents for the band gap in electron volts (eV)

The DFT calculated HOMO–LUMO band gap energy (Fig. 6) showed the role of the substituents in the reactivity of the synthesized compounds. The presence of two electron donating hydroxyl groups in compound **7** (*E*_g_ = 2.242 eV) decreases its relative reactivity to its structurally analogous compound **8** (1.795 eV). The effect of substituent difference for compound **8** (no NO_2_), **9** (one NO_2_) and **11** (two NO_2_) was also observed in their band gap energies (1.795 eV, 2.095 eV and 2.181 eV, respectively).

The wave function distribution for HOMO and LUMO orbitals of compounds **8** and **11** is from uniform (in **8**) distribution to the central part of the molecule (in **11**) as the number of –NO_2_ substituent increases. The localization of electron density is concentrated to the thiazole part of the molecules, decreasing the uniform localization as the number of NO_2_ increases from compound **8** to compound **11** inferring the potential interaction part of the compounds is the thiazole part.

### Molecular electrostatic potential

**Fig. 7:**
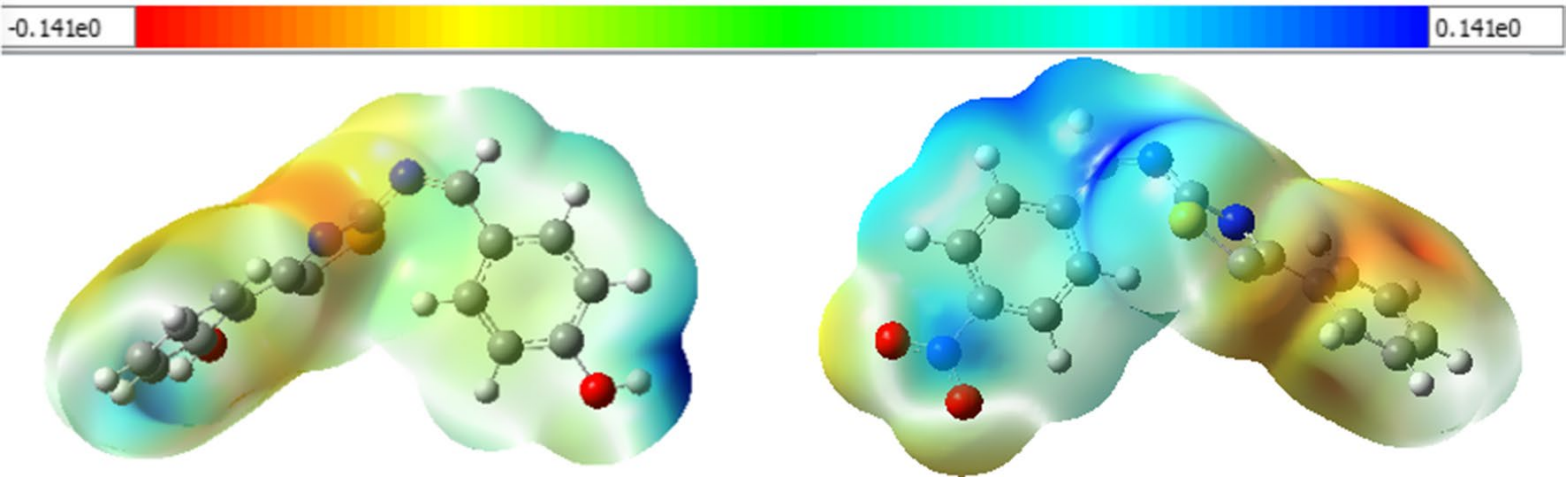
Molecular electrostatic potential map of compound **7** (left) and **10** (right). Negative regions are represented by red, orange, and yellow colors with a decrease in electrostatic potential (yellow → red), whereas positive regions are illustrated by green and blue colors

The molecular electrostatic potential (MEP) surfaces of the synthesized compounds (**7–11**) were plotted from the optimized structures of the compounds at the same level of theory. The MEP maps of the synthesized compounds (**7** and **10**) are presented in Fig. 7 and Additional file 1: Figs. S15–S17. The most negative electrostatic potential region of compound **7** is mainly localized over the thiazole, over the phenyl part of the thiazole for **8** and **10**, whereas on the thiazole and nitro parts of the phenyl thiazole unit of **9**, and the nitro part of phenyl thiazole of compound **11**. These MEP results suggest that the specified regions are the possible binding sites. The dipole moments of the synthesized compounds are found to project to the regions where electrostatic potential region is localized (Additional file 1: Fig. S17).

### Natural atomic charge analysis

The natural atomic charges (NAC) were calculated using B3LYP-GD3/6–311++G(d,p) level of calculation for compounds **7** and **10** (Additional file 1: Fig. S14). The synthesized compounds have nitrogen and sulfur heteroatoms. The oxygen atom of the hydroxy substituents in **7** bear the highest negative charge for the phenyl and benzyl oxygen atoms (− 0.677 and − 0.673, respectively). The thiazole nitrogen of **7** and **10** have the highest negative charges (− 0.53 and − 0.548) relative to the imine nitrogen (− 0.493 and − 0.413), respectively. The sulfur atom of the thiazole unit bears positive natural atomic charge of 0.331 and 0.379, respectively, for compounds **7** and **10**. These charge analyses suggest the presence of charge transfer from sulfur to methine of the thiazole part of the compounds.

### Molecular docking score analysis

**Fig. 8:**
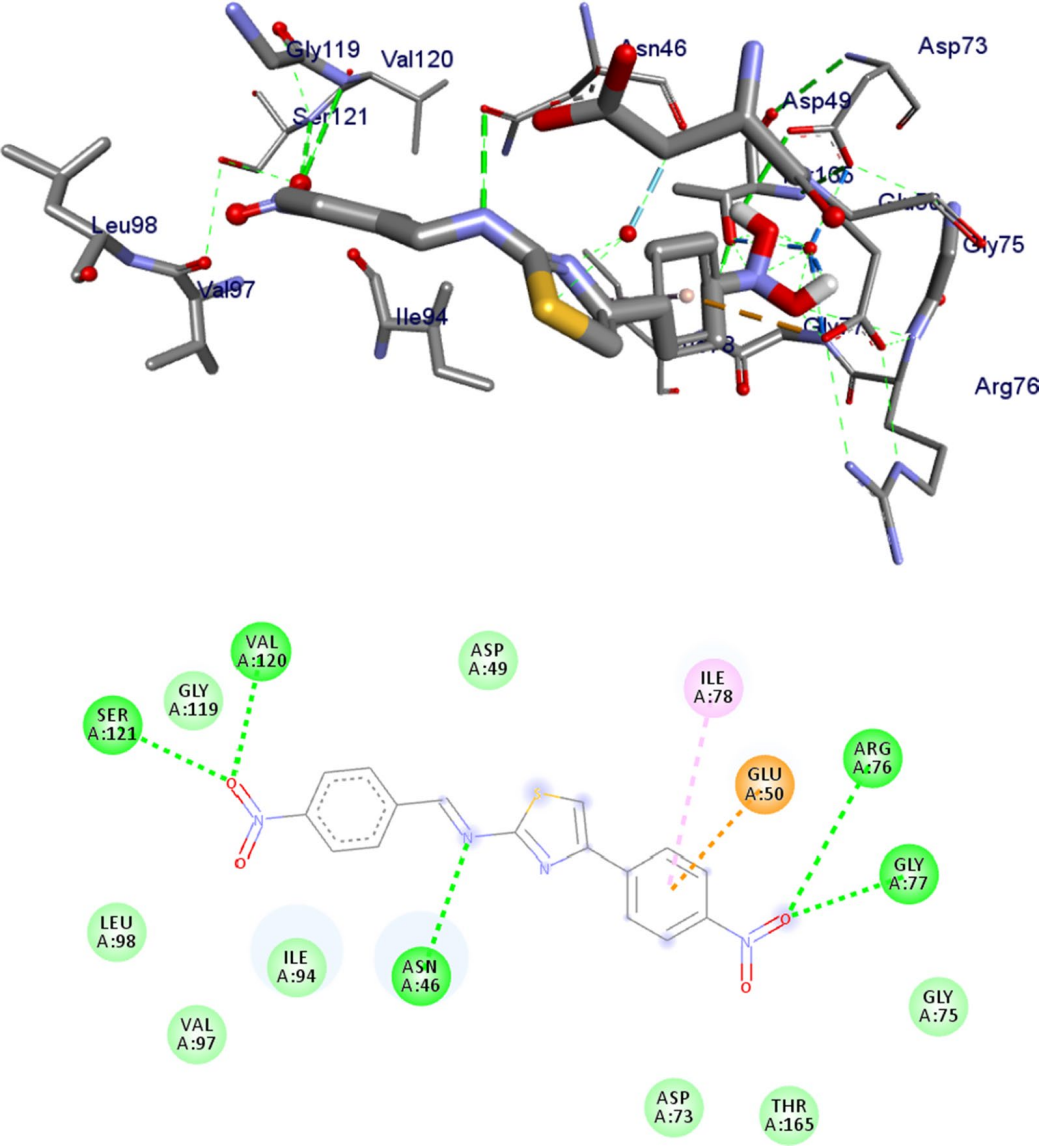
Possible binding interaction of compound **11** against *E. coli* DNA gyrase B (PDB ID: 6F86)

**Fig. 9:**
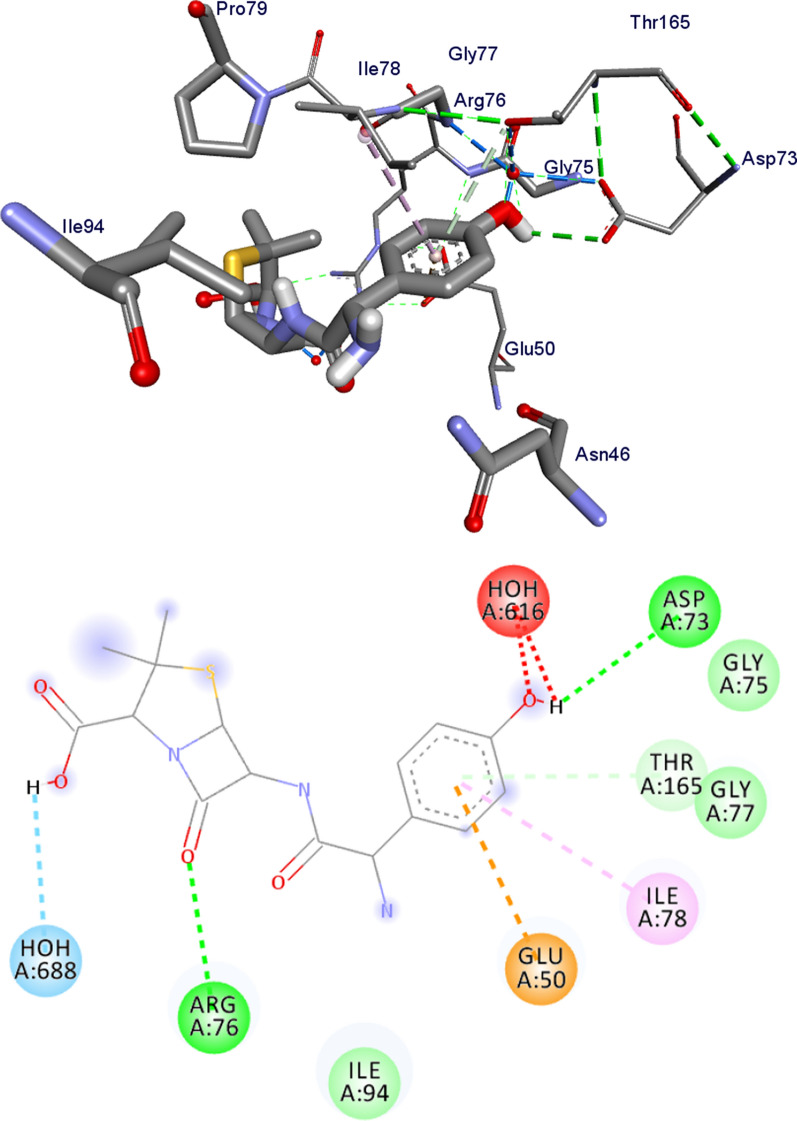
Possible binding interaction of amoxicillin against *E. coli* DNA gyrase B (PDB ID: 6F86)

Most antibacterial agents act by targeting key components of bacterial metabolism: cell-walls, DNA-directed RNA polymerase, protein synthesis and modification, enzymes and DNA gyrase B [63, 73]. To further support the in vitro antibacterial and antioxidant activities of the compounds, molecular docking studies of the five synthesized novel compounds with the binding sites of *E. coli* DNA gyrase B (PDB ID: 6F86) [57] were performed in order to predict the protein–ligand interactions. The results are presented in Figs. 8 and 9 and Additional file 1: Figs. S18–S21. The results showed that the compounds have high binding affinity (− 7.5 to − 6 kcal/mol). The higher binding affinity of compounds **9** and **11** (− 6.9 and − 7.5 kcal/mol, respectively) compared to the standard drug amoxicillin (− 6.1 kcal/mol) suggests that the compounds are promising antibacterial agents against *E. coli* (Table 6) [71]. The higher binding affinity obtained for compounds **9** and **11** is because the compounds have similar residual interactions profile with the amino acid residues Ile-78 (Table 6). Moreover, compounds **7** and **8** have similar residual amino acid (Ala-4, Asp-73, Glu-50, Ile-78 and Thr-165) interactions and comparable binding affinities (Additional file 1: Figs. S18, S19). The hydrogen bond interactions of compound **11** with Asn-46, Val-120, Ser-121 and residual hydrophobic interaction with Glu-50, Ile-78 (Fig. 8; Additional file 1: Figs. S18–S21) also indicated that compound **11** has lower binding affinities, mainly due to the strong electron withdrawing ability of the nitro group of the compound [74]. The findings obtained from the molecular docking analysis (large negative binding affinity) further support the experimental and DFT results (large dipole moment). The overall analysis showed that all the compounds, except **8**, are active against *E. coli* (Table 2). Comparison with the positive control also suggests that the synthesized compounds are found to be promising antibacterial agents compared to amoxicillin.

**Table 6:** Molecular docking affinities of the synthesized compounds against *E. coli* DNA gyrase B (PDB ID: 6F86)

Compounds	Affinity (kcal/mol)	H-bond	Residual amino acid interactions	
			hydrophobic/π-cation/π-anion/π-alkyl interactions	Van-der Walls interactions
**7**	− 6.2	–	Asn-46, Glu-50, Asp-73, Ile-78, Ala-47, Thr-165, Val-167	Val-43,
**8**	− 6.0	Asn-46	Asp-73, Glu-50, Ile-94, Ile-78, Ala-47, Thr-165	Val-43, Val-167
**9**	− 6.9	Glu-50, Ile-94	Arg-76, Asn-46, Ile-78	Gly-77, Gly-75, Thr-165, Val-97, Val-120, Ser-121
**10**	− 6.6	Asn-46, Glu-50	Asp-73, Thr-165, Ala-47, Ile-78	Val-43, Val-167, Ile-94
**11**	− 7.5	Val-120, Asn-46, Ser-121	Glu-50, Ile-78	Asp-73, Asp-49, Gly-77, Gly-75, Thr-165, Val-97, Leu-98, Gly-119
**Amoxicillin**	− 6.1	Asp-73, Arg-76, Gly-77, Thr-165	Glu-50, Ile-78	Gly-77, Gly-75

### Molecular docking against human peroxiredoxin 5

The molecular docking analysis of the synthesized compounds was also carried out to investigate their binding and interaction patterns with human peroxiredoxin 5 (PDB ID: 1HD2) [58]. The in silico study of compounds **7**–**11** showed minimum binding affinities within the binding pocket of human peroxiredoxin 5, ranging from − 5.0 to − 5.3 kcal/mol (Additional file 1: Table S4, Figs. S22–S27). Compound **7** (− 5.3 kcal/mol) displayed the higher binding affinity values compared to ascorbic acid (− 4.9 kcal/mol) (Additional file 1: Figs. S22–S27). As discussed above, the higher antioxidant activity of compound **7** is because of the high energy of HOMO. This is in good agreement with the experimental findings (vide supra) and suggests that compound **7** has potentially good antioxidant activity.

## Conclusions

In the present study, we reported conventional and green synthesis of five thiazole-based Schiff base derivatives together with their ADMET profile predictions, DFT calculated results, molecular docking study against *E. coli* DNA gyrase B and human peroxiredoxin 5, in vitro antibacterial and antioxidant activities. The structures of the synthesized compounds were determined using combined spectroscopic techniques (UV-Vis, FTIR, ^1^H NMR and ^13^C NMR). The results were further supported by DFT calculations. Among the synthesized compounds, compound **11** displayed a promising biological activity. Compounds **7** and **9** showed better DPPH radical scavenging potency. The molecular docking results indicated that compounds **9** and **11** exhibited good interaction with DNA gyrase B. The experimental and computational studies on the antibacterial and antioxidant activities are in good agreement. Compound **7** displayed enhanced minimum binding affinity against human peroxiredoxin 5 compared to ascorbic acid. In silico pharmacokinetics studies showed that all synthesized compounds are biologically significant obeying Lipinski rule of 5. The in silico cytotoxicity predictions revealed that the LD_50_ values of the synthesized compounds are class three (50 ≤ LD_50_ ≤ 300). The experimental and computational results of the present study also suggest that compound **11** is a promising antibacterial agent against *E. coli*, whereas compound **7** was found to possess promising antioxidant potentials. However, further study is recommended to synthesize a series of related thiazole-based Schiff base derivatives for functional group inclusions, structure activity relationship, and in vivo toxicity studies to develop lead antioxidant and antibacterial agents.

## Supplementary Information

**Additional file 1:** Spectral data of the synthesized compounds (^1^H NMR, ^13^C NMR, FTIR and UV–Vis spectra), comparison of experimental and calculated absorption spectra, Autodock Vina conformations of compounds **7**—**11** against *E. coli* DNA gyrase B, NAC, MEP and human peroxiredoxin 5 binding domains, DFT optimized geometries (xyz files), and QM descriptors are included within the additional files.

## Data Availability

The datasets supporting the findings of this article are all presented in the main manuscript. Additional data which further support the findings are presented in the Additional files.
