# Supplementary material for: Synthesis, antibacterial and antioxidant activities of Thiazole-based Schiff base derivatives: a combined experimental and computational study
Source: BMC Chem. 2021 Dec 23;15(1):67. doi: 10.1186/s13065-021-00791-w (PMC8697436; doi:10.1186/s13065-021-00791-w)
Supplement: Supplementary file 1 — Additional file 1: Spectral data of the synthesized compounds (1H NMR, 13C NMR, FTIR and UV–Vis spectra), comparison of experimental and calculated absorption spectra, Autodock Vina conformations of compounds 7—11 against E. coli DNA gyrase B, NAC, MEP and human peroxiredoxin 5 binding domains, DFT optimized geometries (xyz files), and QM descriptors are included within the additional files. [file 13065_2021_791_MOESM1_ESM.pdf]

Synthesis, Antibacterial and Antioxidant Activities of Thiazole Based Schiff Base Derivatives: A Combined Experimental and Computational Study

Fitsum Lemilemu[1*], Mamaru Bitew[1], Taye B. Demissie[2], Rajalakshmanan Eswaramoorthy[3], Milkyas Endale[1*]

[1] Department of Applied Chemistry, Adama Science and Technology University, P.O. Box 1888, Adama, Ethiopia.

[2] Department of Chemistry, University of Botswana, Notwane Rd, P/bag UB 00704 Gaborone, Botswana. [3]Department of Biomaterials, Saveetha Dental College and Hospital, Saveetha University, Chennai, India.

Correspondence: milkyas.endale@astu.edu.et, fitsumlemilemu@gmail.com

Table of Contents

1.1 Characterization of synthesized compounds (**7-11**). 2

1.2 NMR spectral data characterization of synthesized compounds (**7-11**). 2

1.3 The experimental/computational maximum TD-DFT/UV-Vis absorption bands of compound **8** and **9**. 14

1.4 The DPPH scavenging activity and IC_{50} of synthesized compounds (**7-11**)......................16

1.5 In Silico pharmacokinetics and toxicity profile of synthesized compounds (**7-11**). 18

1.6 Natural atomic charge analysis of synthesized compounds (**7-11**). 19

1.7 Molecular electrostatic potential of compound **8**, **9** and **11**. 21

1.8 Molecular docking affinities analysis of synthesized compounds **7**, **8**, **9** and **10** against DNA Gyrase B. 23

1.9 Molecular docking affinities analysis of synthesized compound (**7-11**) against human peroxiredoxin 5..28

xyz coordinates of the B3LYP-GD3/6-311++G(d,p) optimized geometries 35

References 46

1.1 Characterization of synthesized compounds (7-11).

Table S1 Physical properties of compounds **(5-11)**

**Compound**	**Molecular Formula**	**MW (calculated) (g/mol)**	**Melting Point (^{0}C)**	**Physical State**	**Color**	**% Yield**	**R_f Value**
**5a**	$C_9H_8N_2S$	176.24	144-146	Solid	White	99.2	0.66 (Hex: EA 4:1)
**5b**	$C_9H_8N_2OS$	192.24	92-94	Powder	Red	55	0.33 (Hex: EA 7:3)
**5c**	$C_9H_7N_3O_2S$	221.24	226-228	Solid	Yellow	98.64	0.62 (Hex: EA7:3)
**7**	$C_{16}H_{12}N_2O_2S$	296.34	152-154	Solid	Red	68.83	0.8 (Hex: EA 1:1)
**8**	$C_{16}H_{12}N_2S$	264.34	170-172	Solid	White	77.3	0.77 (Hex: Et 1:2)
**9**	$C_{16}H_{11}N_3O_3S$	325.34	190-192	Powder	Pale orange	69.3	0.86 (Hex: EA 7:3)
**10**	$C_{16}H_{11}N_3O_2S$	309.34	148-150	Powder	Green	84.6	0.67 (Hex: EA 1:4)
**11**	$C_{16}H_{10}N_4O_4S$	354.34	188-190	Solid	Yellow	83.3	0.44 (Hex: EA 2:1)

Notice: Hex Hexane, EA Ethyl acetate

Note that, the yield of compound **5**b was found to be small compared to other intermediates (**5**a and **5**c). This is because of the electron donating effects of the hydroxyl group on position 2 of the phenyl ring which enhances the stability of the reactant (2-hydroxoacetophenone) via resonance effect of the hydroxyl group of phenyl ring. The lone pair of electrons on the oxygen can be donated back into the phenyl ring by resonance, giving rise to an additional resonance structure, so the 2- hydroxyl substituents are the electron donating group reduced the yield of reaction [1].

1.2 NMR spectral data characterization of synthesized compounds (7-11).

Compound 7 (0.53g, R_f value of 0.8 using Hex: EA 1:1 as eluent) were given in Table S1. UV-Vis λ_{max} (DMSO) =260 nm. Its ^{1}H NMR spectrum (DMSO-d_6, 400 MHz, δ in ppm) showed

a singlet peaks at *δ* 9.79 (s, 1H, H-13) and *δ* 7.86 (s, 1H, H-8) attributed to imine bond and thiazole ring protons, respectively [2]. The presence of AA'XX' spin system at *δ* 7.76 (d, 1H, H-18, *J* = 8 Hz), 7.78 (d, 1H-16, *J* = 8 Hz), 8.15 (d, 1H, H-15, *J* = 12 Hz), 8.19 (d, 1H, H-19, *J* = 12 Hz) suggest the presence of para substituted phenyl ring. The presence of phenol protons was observed at 9.49 (s, 1H, H-21). A disubstituted phenyl ring was evident from peaks at *δ* 7.48 (d, 1H, H-6, *J* = 8 Hz), 6.86(d, H-2, *J* = 8 Hz) 6.84(m, 1H, H-1), 7.46 (d, 1H, H-3, *J* = 8 Hz) and singlet aromatic hydroxyl proton at δ 9.62 (s, 1H, H-20). Its ^{13}C NMR (100 MHz, DMSO-d_6), *δ* in ppm showed fourteen peaks at *δ* 169.4 (C-13), 162.4 (C-17), 158.4(C-4) 156.4(C-10) ,152.8 (C-14), 141.8 (C-5), 135.9(C-15, 19), 132.2 (C-4,6), 131.3 (C-7), 129.2(C-2), 122.8 (C-1), 121.8 (C-3), 120.5 (C-16,18) and 115.8 (C-8). All the spectral data displayed are in good agreement with the proposed structures are in Figure S1 and S2.

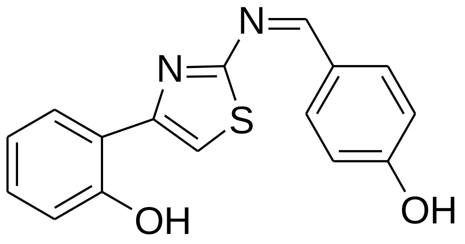

7

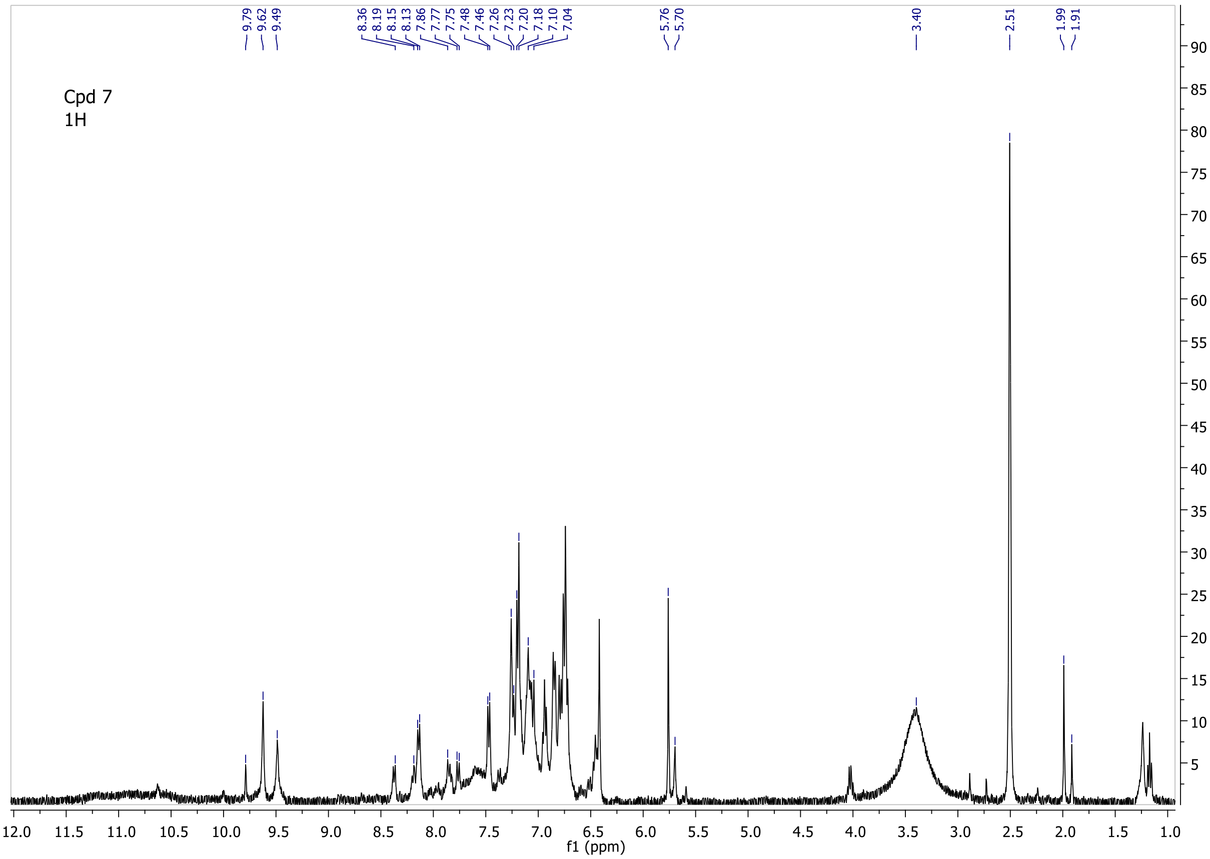

Figure S1. ^{1}H NMR (400 MHz, DMSO-d_6) spectrum of compound **7**.

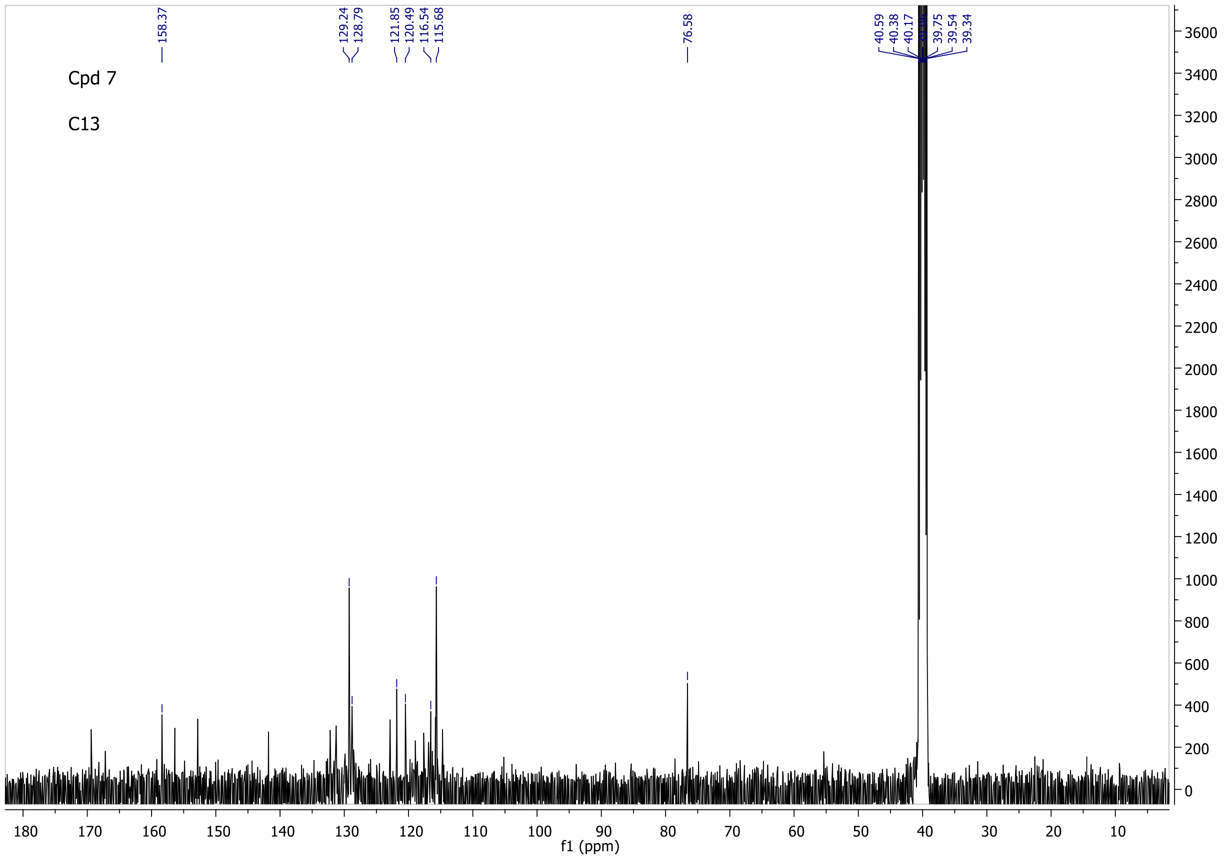

Figure S2. ^{13}C NMR (100 MHz, DMSO-d_6) spectrum of compound **7.**

Compound 8 (0.53 g, R_f value of 0.77 using Hex: EA 1:2 as eluent), melting point 170-172°C. Its UV-Vis λmax (MeOH) =214 nm. ^{1}H NMR spectrum (DMSO-d_6 400 MHz, δ in ppm) showed two singlet peaks attributed to imine and thiazole ring protons at δ 7.64 (s, H-13) and 7.30 (s,1H, H-8), respectively. The presence of two mono substituted phenyl rings were evident at δ 7.39 (dd, 1H, H-4,6, J = 8 Hz),7.37 (dd,1H, H-1,3, J = 8 Hz), 7.35 (m, 1H, H-2) and 7.28 (dd, H-15, J = 8 Hz), 7.24 (dd, H-19, J = 8 Hz),7.22(dd, H-16, J = 8 Hz), 7.20 (dd,1H, H-18, J = 8 Hz) and 7.18 (m, H-17). Its ^{13}C NMR (100 MHz, DMSO-$d_{6,}$ δ in ppm) displayed twelve peaks at δ 167.3 (C-13), 149.7(C-10), 144.4 (C-7),136.6 (C-5),133.6 (C-14), 129.3 (C-17), 128.7 (C-15, 19), 128.5 (C-16, 18), 128.4 (C-1, 3), 127.8 (C-2), 125.8 (C-4,6) and 122.8 (C-8). All the spectral data displayed are in good agreement with the proposed structures in Figure S3 and S4. This is also in agreement with previously reported results with less yield [3].

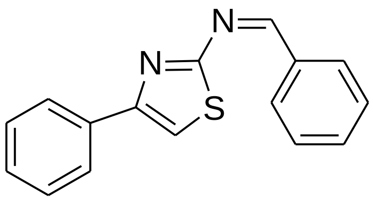

8

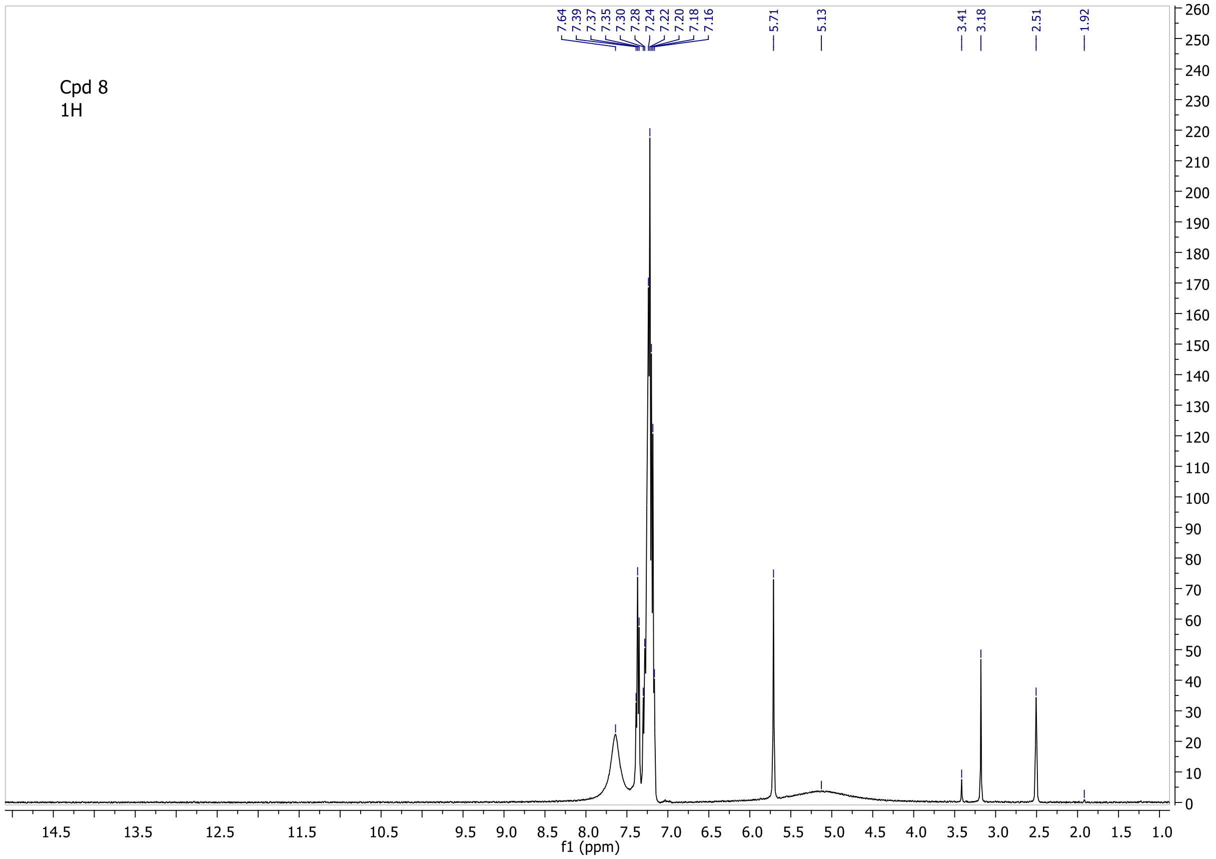

Figure S3. ^{1}H NMR (400 MHz, DMSO-d_6) spectrum of compound **8.**

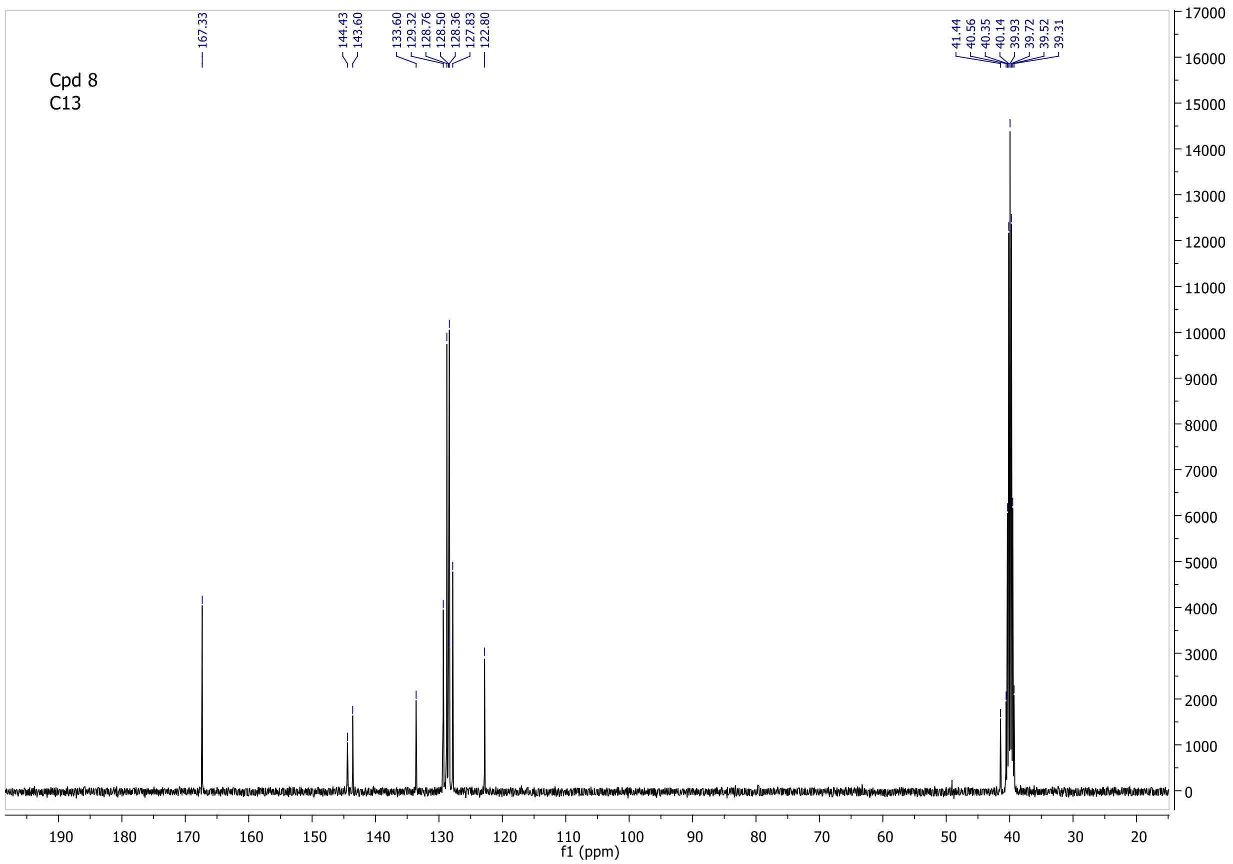

Figure S 4. ^{13}C NMR (100 MHz, DMSO-d_6) spectrum of compound **8.**

Compound 9 (0.507 g, R_f value of 0.86 using Hex: EA 7:3 as eluent), melting point of 148-150°C. UV-Vis λmax (DMSO) =260 nm. Its ^{1}H NMR spectrum (DMSO-d_6 400 MHz, δ in ppm) showed a singlet peak at δ 8.86 (H-13) and 8.3 (H-8) attribute to imine and thiazole ring protons, respectively. The presence of two AA'XX' spin system para-substituted phenyl rings were observed at δ 8.17 (dd, H-1,3, J = 8,12 Hz), 8.12(dd,1H, H-4,6, J = 8,12 Hz) and 7.96 (d,1H, H-15, J = 8 Hz), 7.9 (d, 1H, H-19, J = 8 Hz), 7.69 (d, 1H, H-16, J = 8 Hz), 7.67 (d, 1H, H-18, J = 8 Hz) and the presence of aromatic hydroxyl group at δ 10.02 (s, 1H, H-20). The presence of AA'XX' spin system is clearly evident at δ 9.07 (d, 1H, H-16, 18, J = 8 Hz) and 8.06 (d, 1H, H-15, 19, J = 8 Hz) suggesting a para substituted phenyl ring. Its ^{13}C NMR (100 MHz, DMSO-d_6 δ in ppm) showed twelve peaks at δ 168.1 (C-13), 149.5 (C-10), 147.3 (C-17), 135. (C-7), 128.9 (C-14),128.7(C-5),128.4(C-15,19),127.9(C-1,3),128.7(C-2),128.3(C-4,6),124.2 (C16,18) and 120

(C-8). All the spectral data displayed are in good agreement with the proposed structures are in Figure S5 and S6.

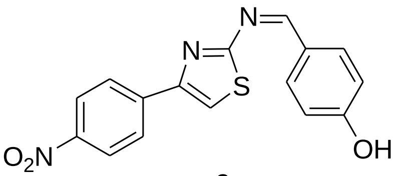

9

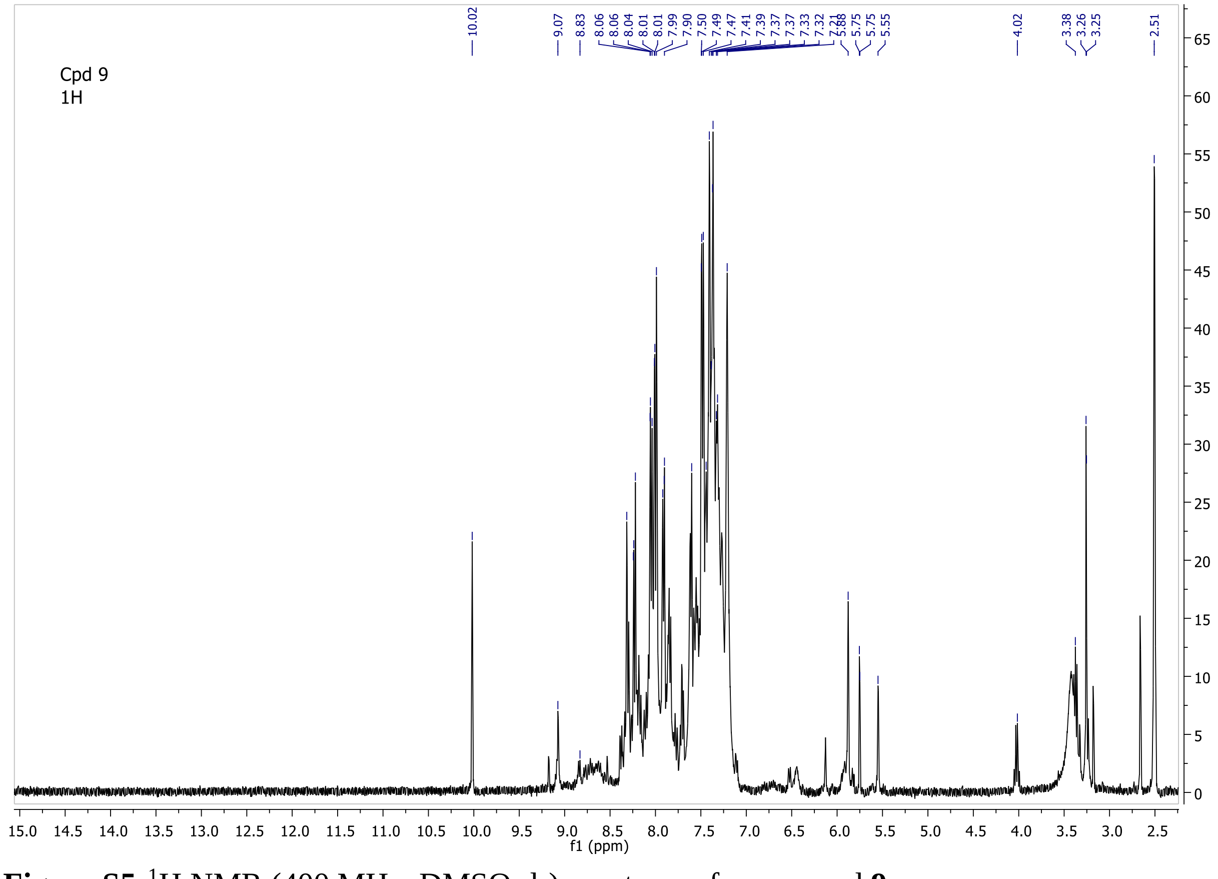

Figure S5. ^{1}H NMR (400 MHz, DMSO-d_6) spectrum of compound **9.**

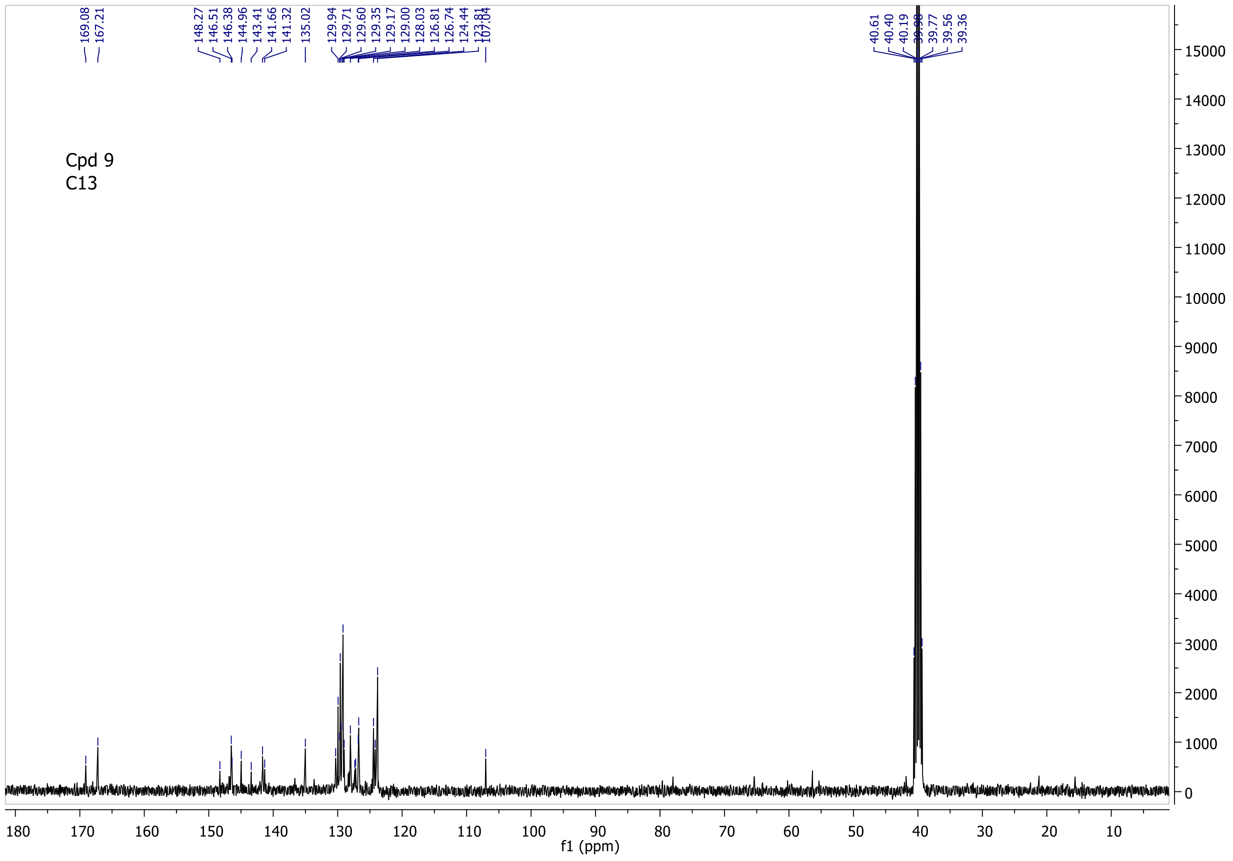

Figure S6. ^{13}C NMR (100 MHz, DMSO-d_6) spectrum of compound **9.**

Compound 10 (0.55g, R_f value of 0.67 using Hex: EA 1:4 as eluent), melting point of 190-192°C. UV-Vis λ_{max} (DMSO) =269 nm. Its ^{1}H NMR spectrum (DMSO-d_6 400 MHz, δ in ppm) showed singlet peaks at δ 7.39 (H-13) and 7.18 (H-8) attributed to imine and thiazole ring protons, respectively. The presence of AA'XX' spin system phenyl ring is clearly evident from peaks at δ 8.24 (d, 1H, H-16, 18, J = 8Hz) and 8.22 (d, 1H, H-15, 19, J = 8Hz). The presence of monosubstituted phenyl ring is evident having from peaks at δ 7.57 (m, 1H, H-2), 7.47 (d, 1H, H-4, 6, J = 8Hz) and 7.45 (dd, 1H, H-1, 3, J = 8Hz). Its ^{13}C NMR (100 MHz, DMSO-d_6) showed twelve peaks at δ 168.1 (C-13), 149.5 (C-10), 147.3 (C-17), 135. (C-7), 128.9 (C-14),128.7(C-5),128.4(C-15,19),127.9(C-1,3),128.7(C-2),128.3(C-4,6),124.2(C16,18) and 120 (C-8). All the spectral data displayed are in good agreement with the proposed structures are in Figure S7and S8. This is also in agreement with previously reported results with less yield [4].

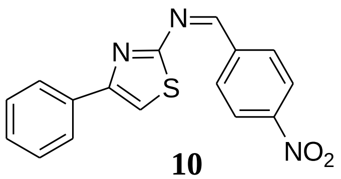

10

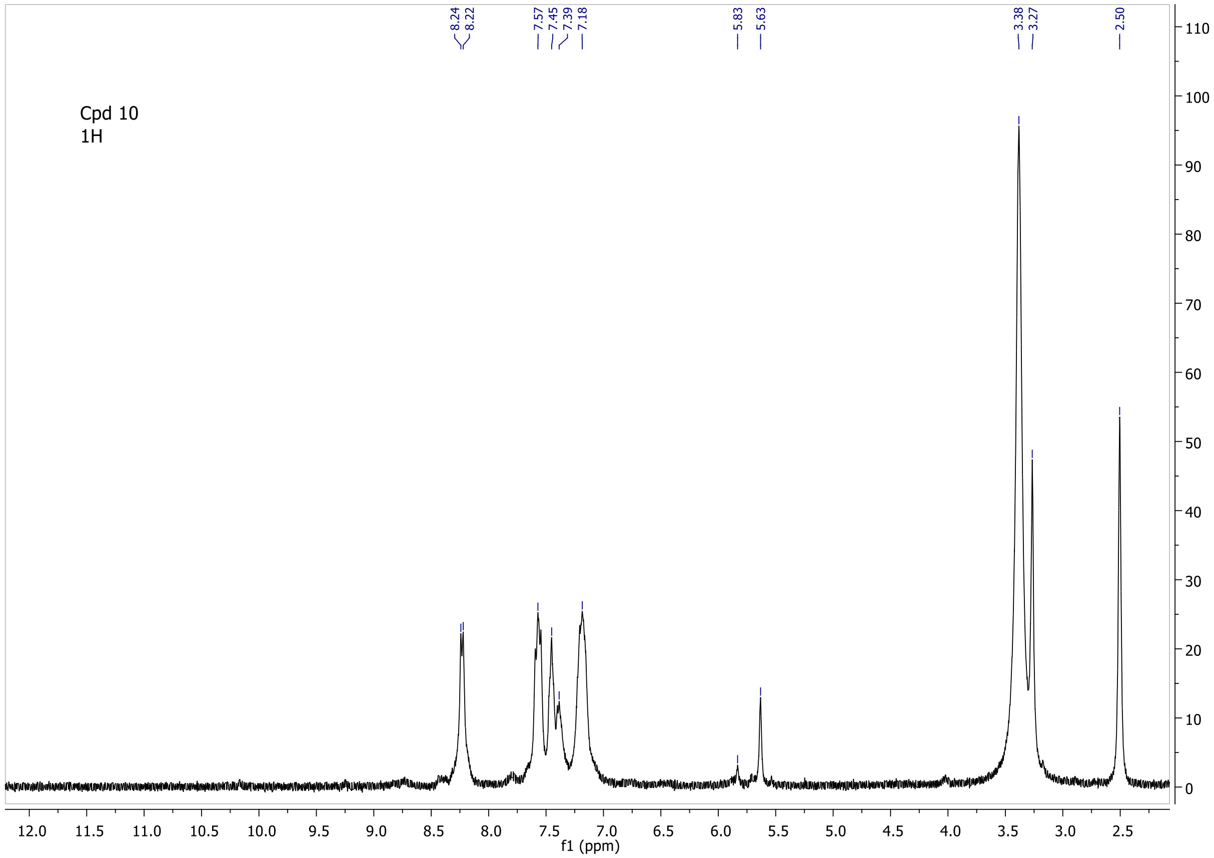

Figure S7. ^{1}H NMR (400 MHz, DMSO-d_6) spectrum of compound **10.**

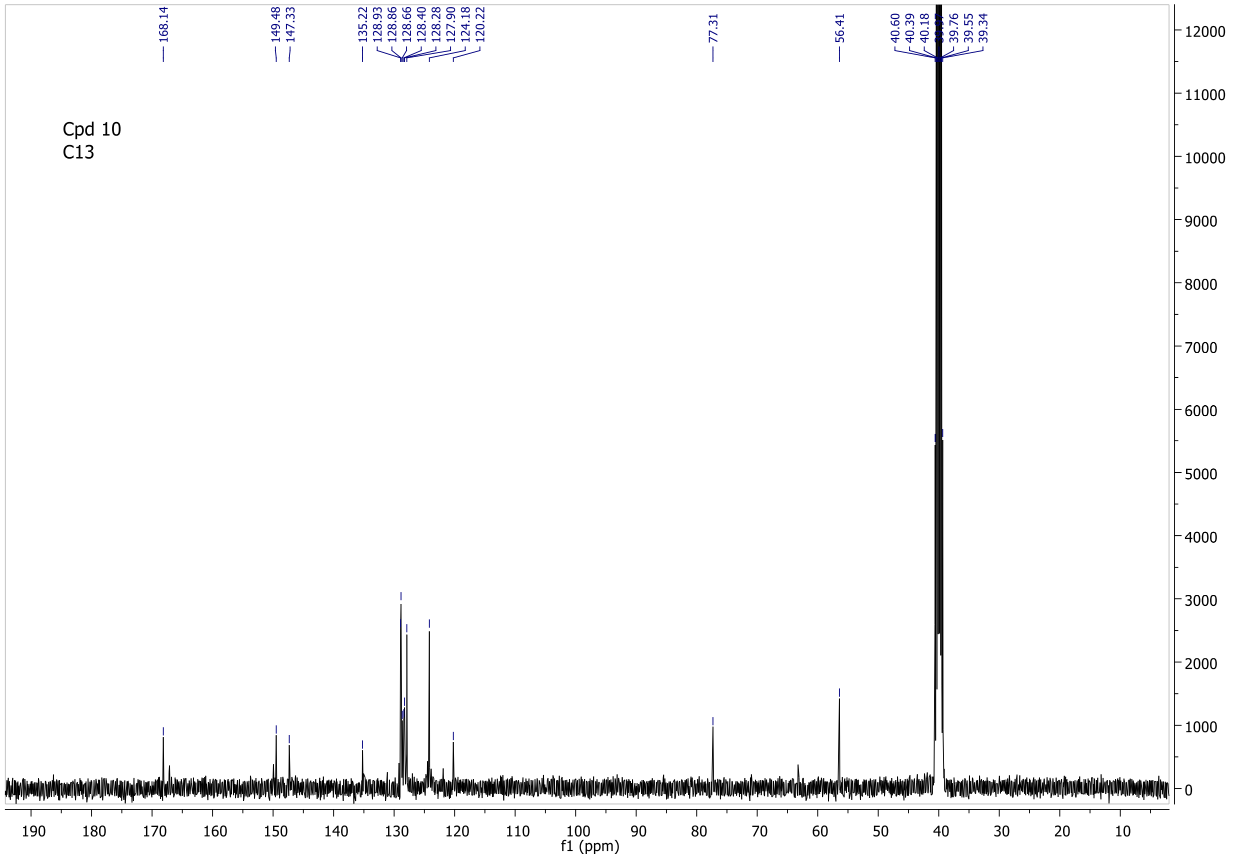

Figure S8. ^{13}C NMR (100 MHz, DMSO-d_6) spectrum of compound **10.**

Compound 11 (0.48 g, Rf value of 0.4 using Hex: EA 2:1 as eluent), melting point 188-190°C. UV-Vis λmax (DMSO) =276 nm; FTIR spectrum peaks of C-H (benzyl) at 2936 cm^{-1}, C=N (imine) at 1611 cm^{-1} that confirmed the synthesis of the designed Schiff base, and peaks from 1384 -1104 cm^{-1} (overlapping peaks of the thiazole nucleus the nitro group), a sharp peak at 608 cm^{-1} (S-C) in Figure S10 (bottom). Compound 11 showed a peak at 3432 cm^{-1} which may be due to moisture absorption [5]. Its ^{1}H NMR spectrum (DMSO-d_6, 400 MHz, δ in ppm) showed singlet peaks at δ 8.88 (H-13) and 8.21(H-8) that belong to imine and thiazole protons, respectively. The presence of two AA'XX' spin system phenyl rings were observed at δ 8.04 (m,1H, H-1,3) ,7.85 (m, 1H, H-16,18), 7.66 (dd, 1H, H-4,6, J= 8,24 Hz) and 7.64 (d,1H, H-15,19, J = 8,24 Hz). Its ^{13}C NMR (100 MHz, DMSO-d_6, δ in ppm) showed twelve peaks at δ 169.1 (C-13), 150.8 (C-10), 148.3 (C-17, C-NO_2), 147.2 (C-2, C-NO_2), 146.3 (C-7), 143.8 (C-14),141.3 (C-5),128.2 (C-15,19), 126.7

(C-4,6), 124.4 (C-16, 18), 123.8 (C-1,3) and 107.0 (C-8). All the spectral data displayed are in good agreement with the proposed structures are in Figure S9 and S10.

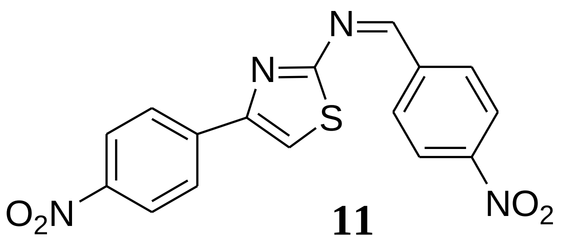

11

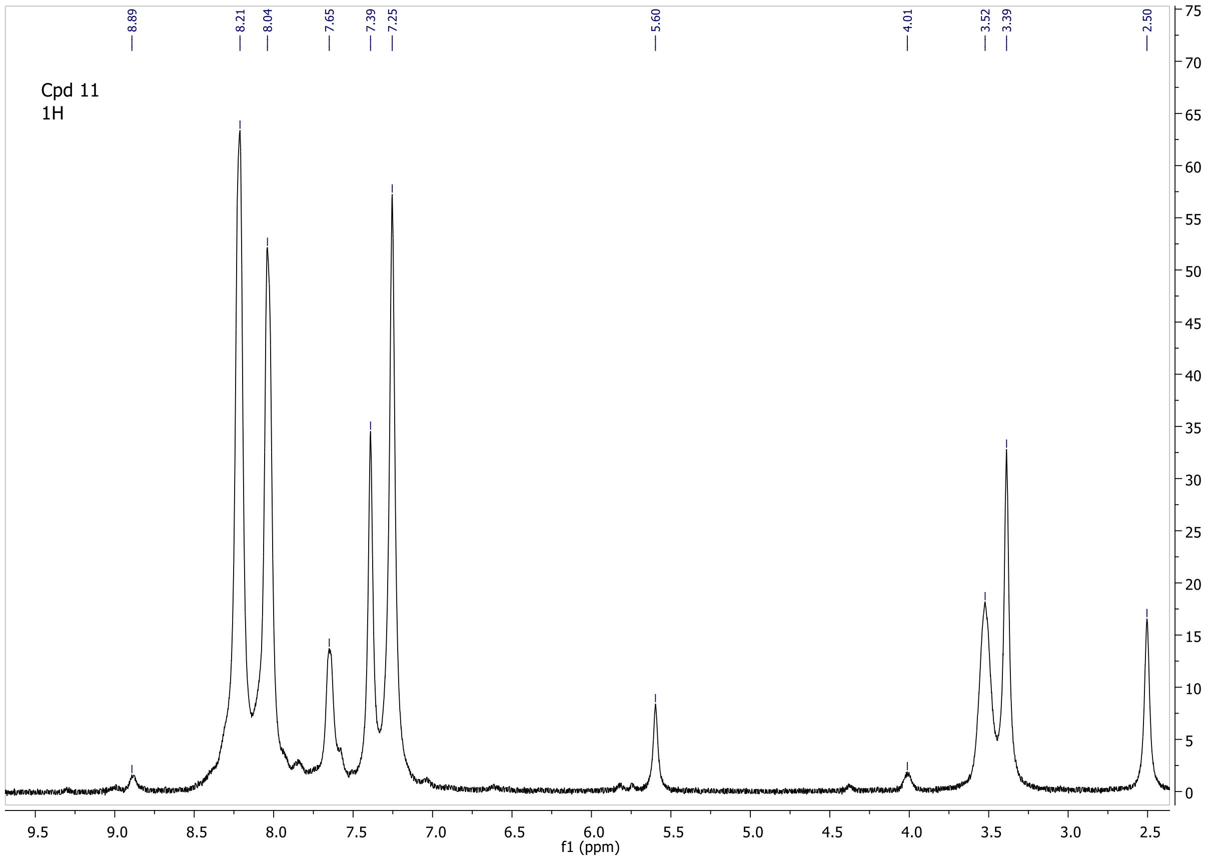

Figure S9.^{1}H NMR (400 MHz, DMSO-d_6) spectrum of compound **11**.

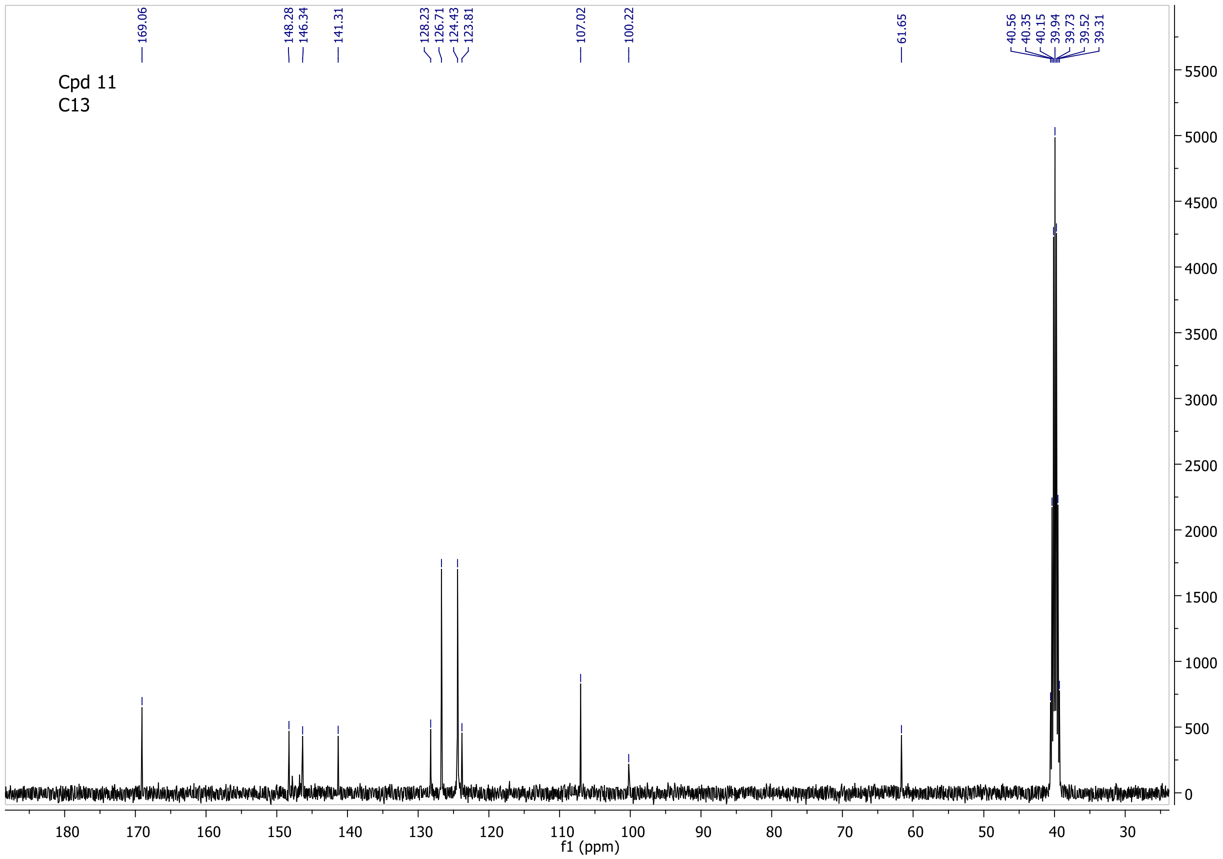
Cpd 11
C13
169.06
148.28
146.34
141.31
128.23
126.71
124.43
123.81
107.02
100.22
61.65
40.56
40.35
40.15
39.94
39.73
39.52
39.31
f1 (ppm)

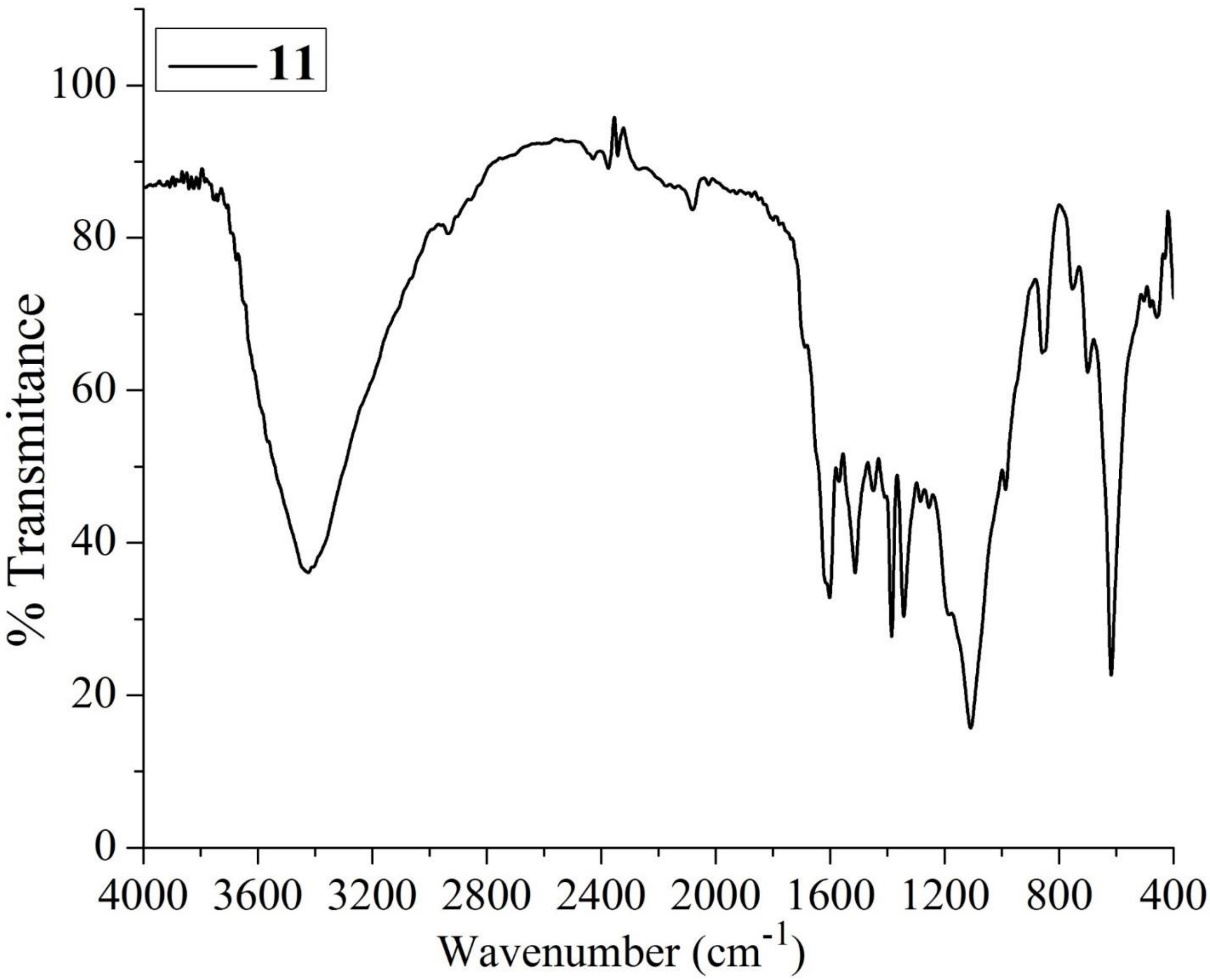

Figure S10. ^{13}C NMR (100 MHz, DMSO-d_6) spectrum of compound 11 top**)** and Ft-IR **(**bottom**).**

1.3 The experimental/computational maximum TD-DFT/UV-Vis absorption bands of compound 8 and 9.

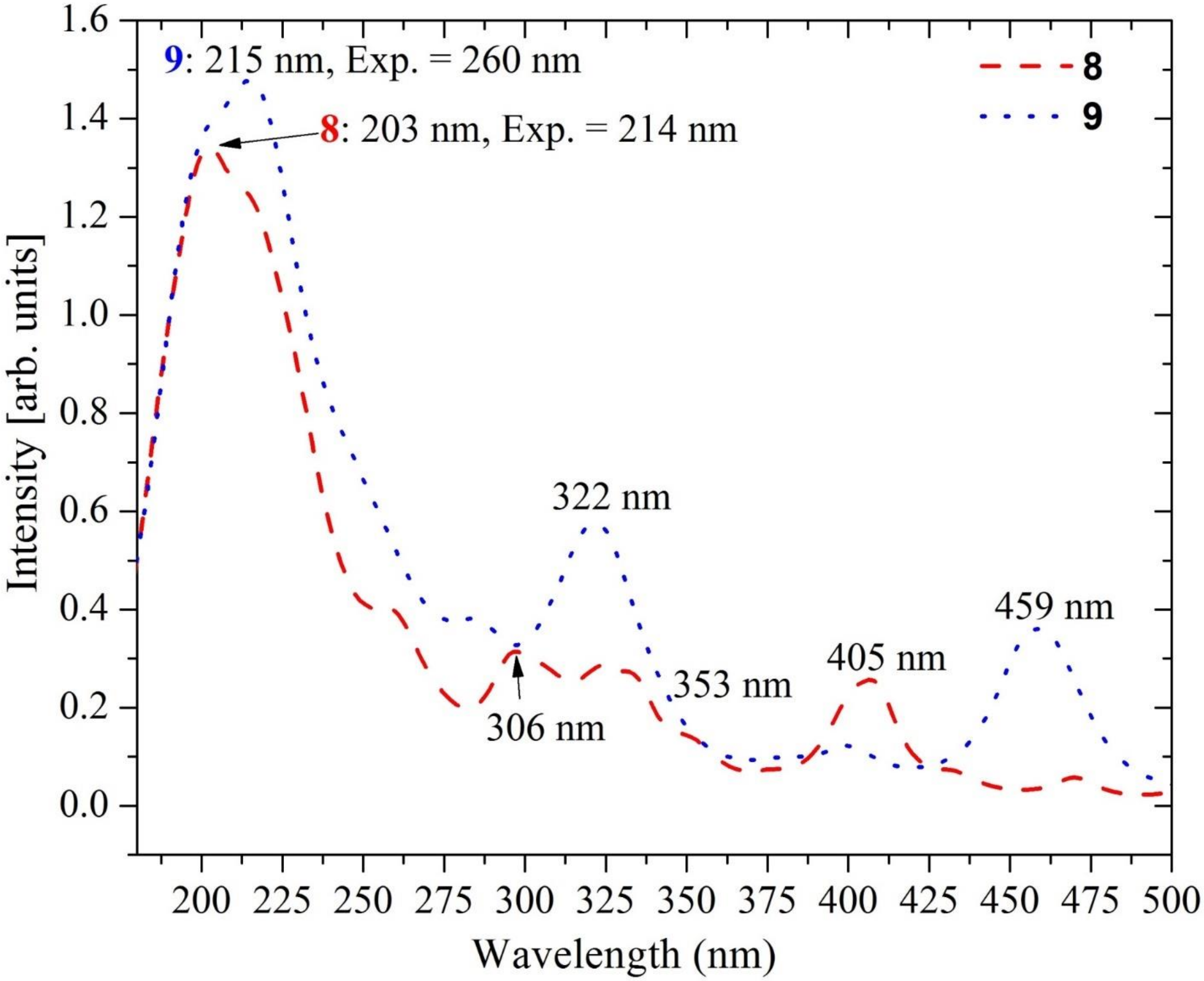

Figure S11. Comparison of the experimental absorption wavelengths with the corresponding B3LYP/6-311++G(d,p) results. The calculated spectra were red-shifted by 30 nm for better comparison with the experimental results.

1.4 The DPPH scavenging activity and IC_{50} of synthesized compounds (7-11).

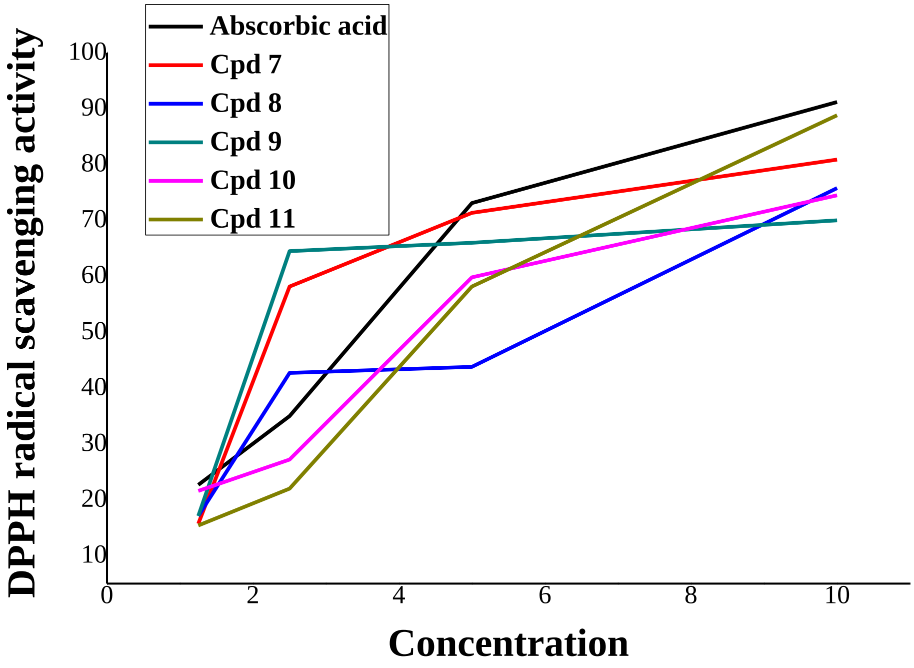

Figure S12. DPPH scavenging activity (%) of synthetized compounds with positive reference L-Ascorbic acid.

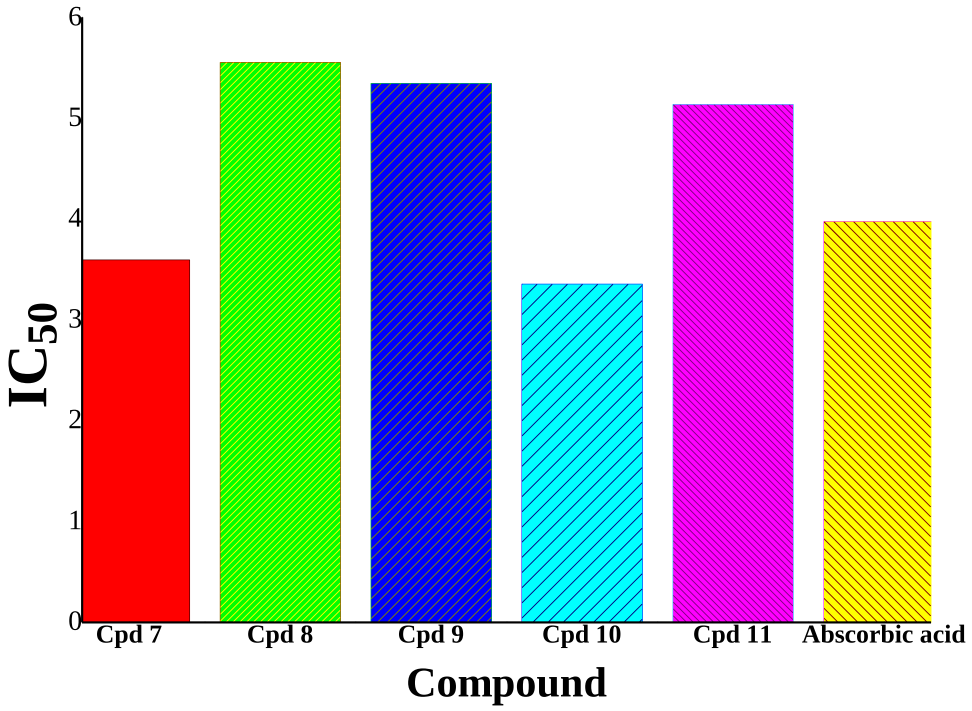

Figure S13. Half maximal inhibitory concentration of synthetized compound (**7-11**) with IC_{50} via L-Ascorbic acid standard.

1.5 *In Silico* pharmacokinetics and toxicity profile of synthesized compounds (7-11).

Table S2 Molecular descriptors for ADME of the synthesized compounds.

Amx = Amoxicillin, GI = Gastro intestinal, BBB = blood brain barrier, P-gp = Protoglycoprotein,

Cpds	GI Absorpti	BBB permeant	Pgp substrate	CYP1A2 inhibitor	CYP2C19 inhibitor	CYP2C9 inhibitor	CYP2D6 inhibitor	CYP3A4 inhibitor	log Kp (cm/s)
7	High	No	No	Yes	Yes	Yes	No	No	-5.59
8	High	Yes	No	Yes	Yes	Yes	No	No	-4.66
9	Low	No	No	Yes	Yes	Yes	No	No	-5.41
10	High	No	No	Yes	Yes	Yes	No	No	-5.29
11	Low	No	No	Yes	Yes	Yes	No	No	-5.45
Amx	Low	No	No	No	No	No	No	No	No

CYPs = Cytochrome family enzymes, Cpds = Compounds.

Table S3 Organ toxicity and toxicological end points of synthesized compounds predicted by Pro Tox II.

Compounds	Hepatotoxicity	Carcinogenicity	Immunotoxicity	Mutagenicity	Cytotoxicity	LD_{50} (mg/Kg)	Toxicity class
7	Active	Active	Inactive	Active	Inactive	300	3
8	Active	Inactive	Inactive	Active	Inactive	300	3
9	Active	Active	Inactive	Active	Inactive	300	3
10	Active	Active	Inactive	Active	Inactive	300	3
11	Active	Active	Inactive	Active	Inactive	300	3
Amx	Inactive	Inactive	Inactive	Inactive	Inactive	15,000	6

1.6 Natural atomic charge analysis of synthesized compounds (7-11).

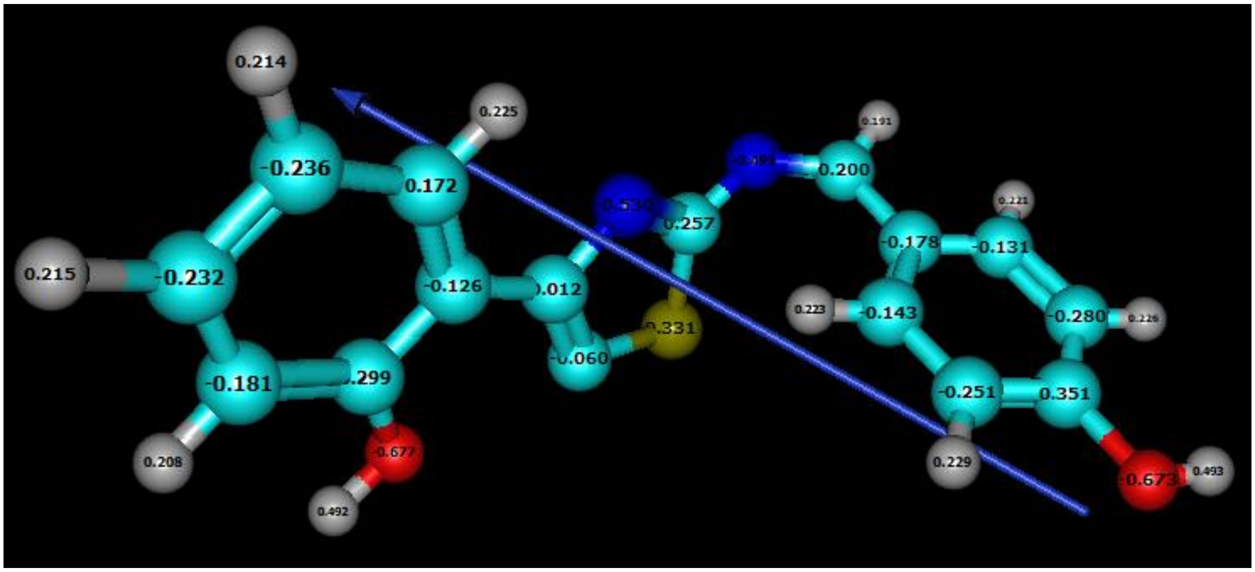

7

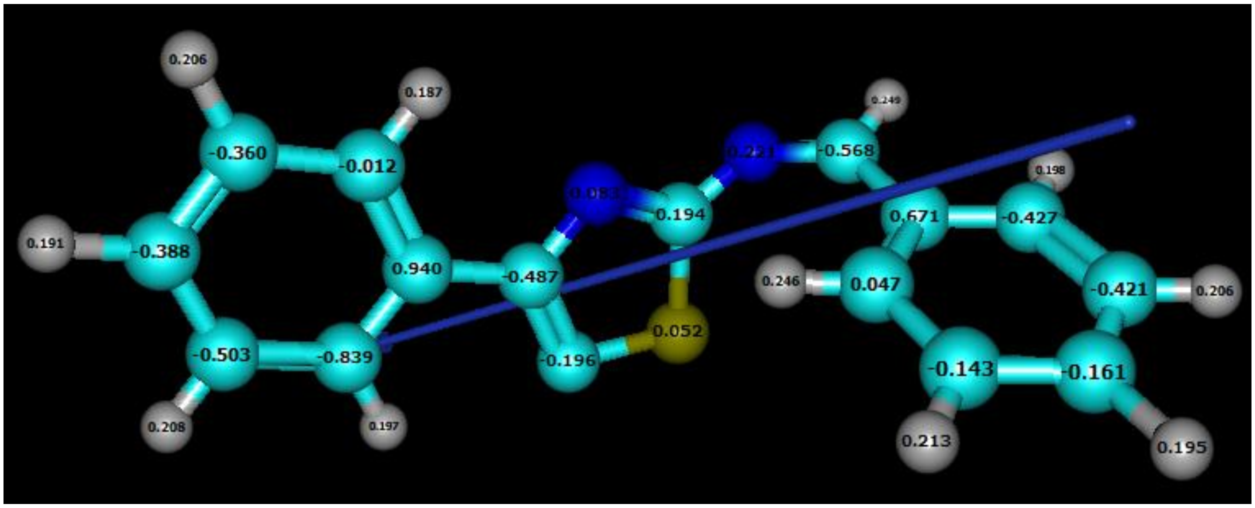

8

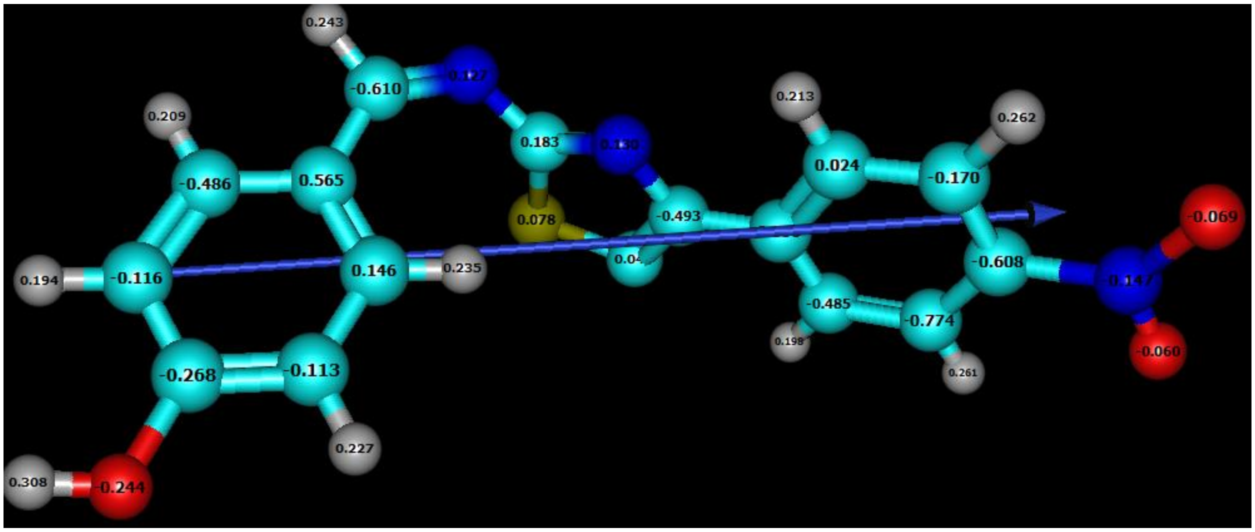
0.243
-0.610
0.209
0.183
0.213
0.262
-0.486
0.565
0.024
-0.170
0.078
-0.493
-0.069
0.194
-0.116
0.146
0.235
-0.608
-0.485
-0.774
0.198
-0.060
-0.268
-0.113
0.261
0.227
0.308
-0.244

9

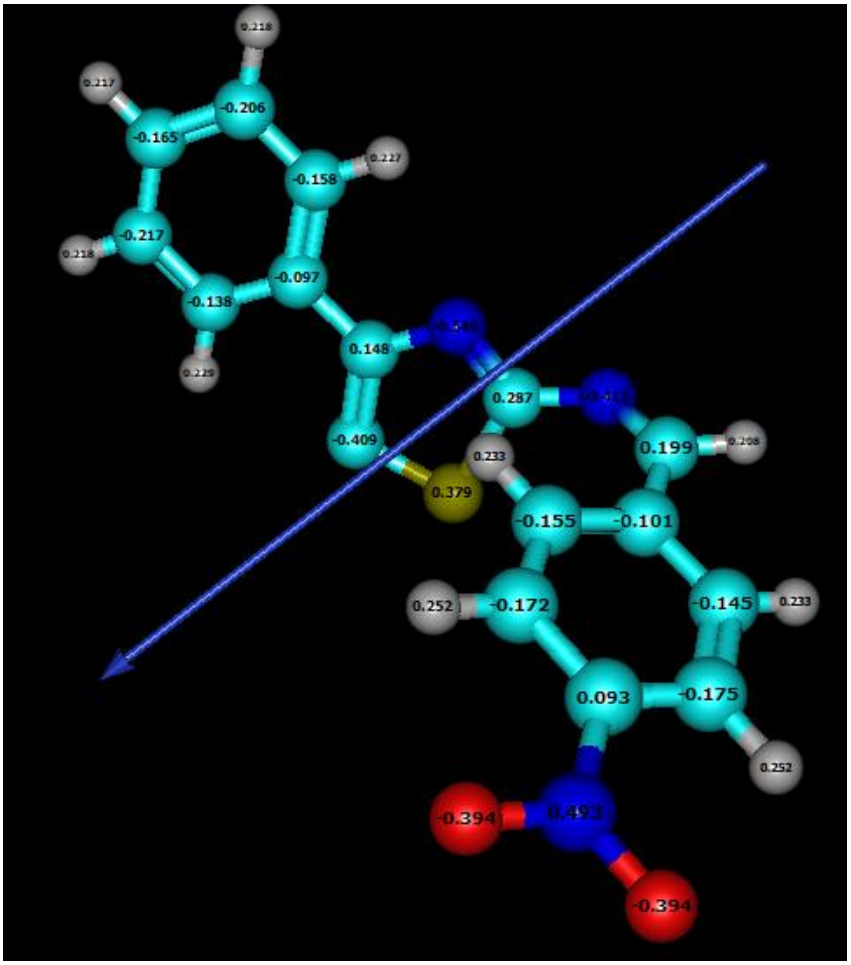
-0.206
-0.165
-0.158
-0.217
-0.097
-0.138
0.148
0.287
0.199
-0.409
0.233
0.379
-0.155
-0.101
0.252
-0.172
-0.145
0.233
0.093
-0.175
0.252
-0.394
0.493
-0.394

10

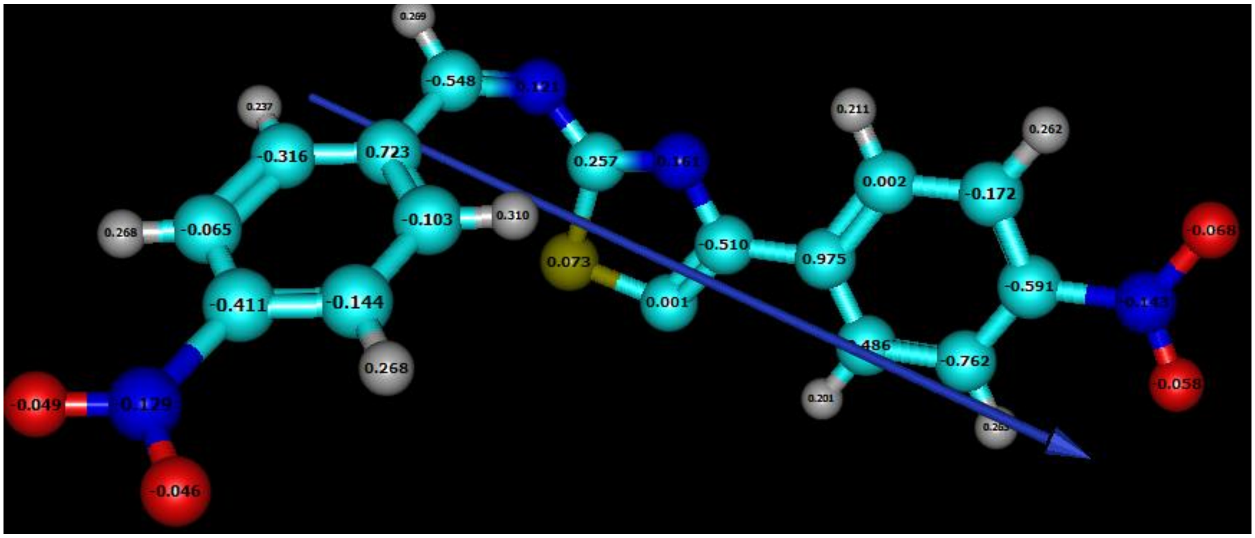

11

Figure S14. The natural atomic charge distribution of from compound **7** (top) to compound **11** (bottom).

1.7 Molecular electrostatic potential of compound 8, 9 and 11.

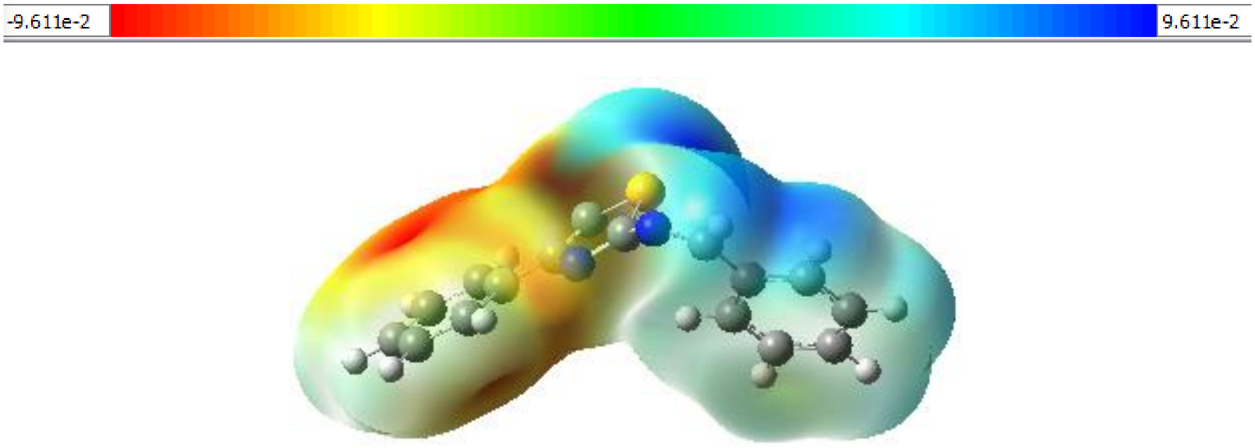

Figure S15. Molecular electrostatic potential map of compound **8**. Negative regions are represented by red, orange and yellow colors, whereas positive regions are illustrated by green and blue colors.

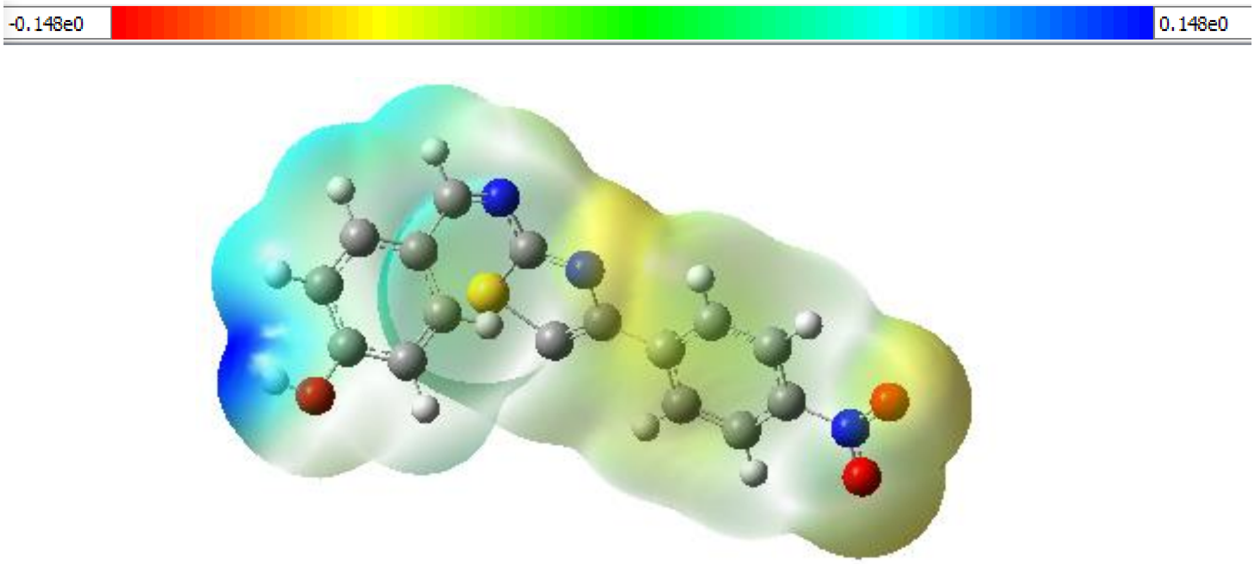

Figure S16. Molecular electrostatic potential map of compound **9.**

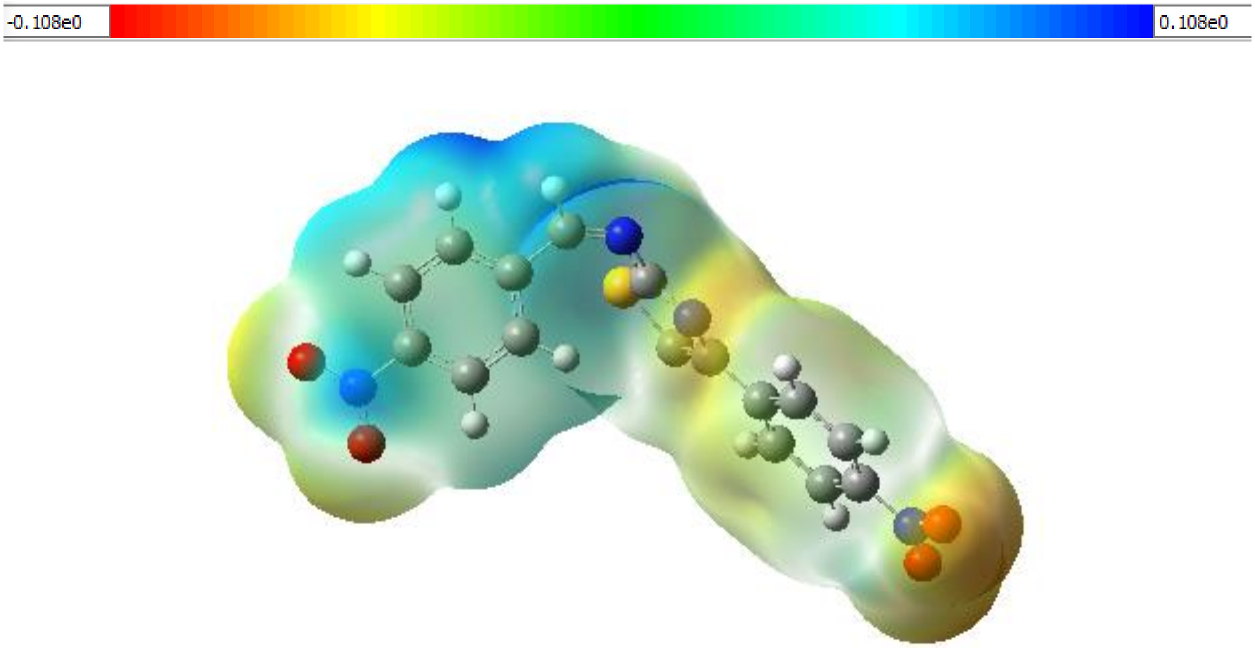

Figure S17. Molecular electrostatic potential map of compound **11.**

1.8 Molecular docking affinities analysis of synthesized compounds 7, 8, 9 and 10 against DNA Gyrase B.

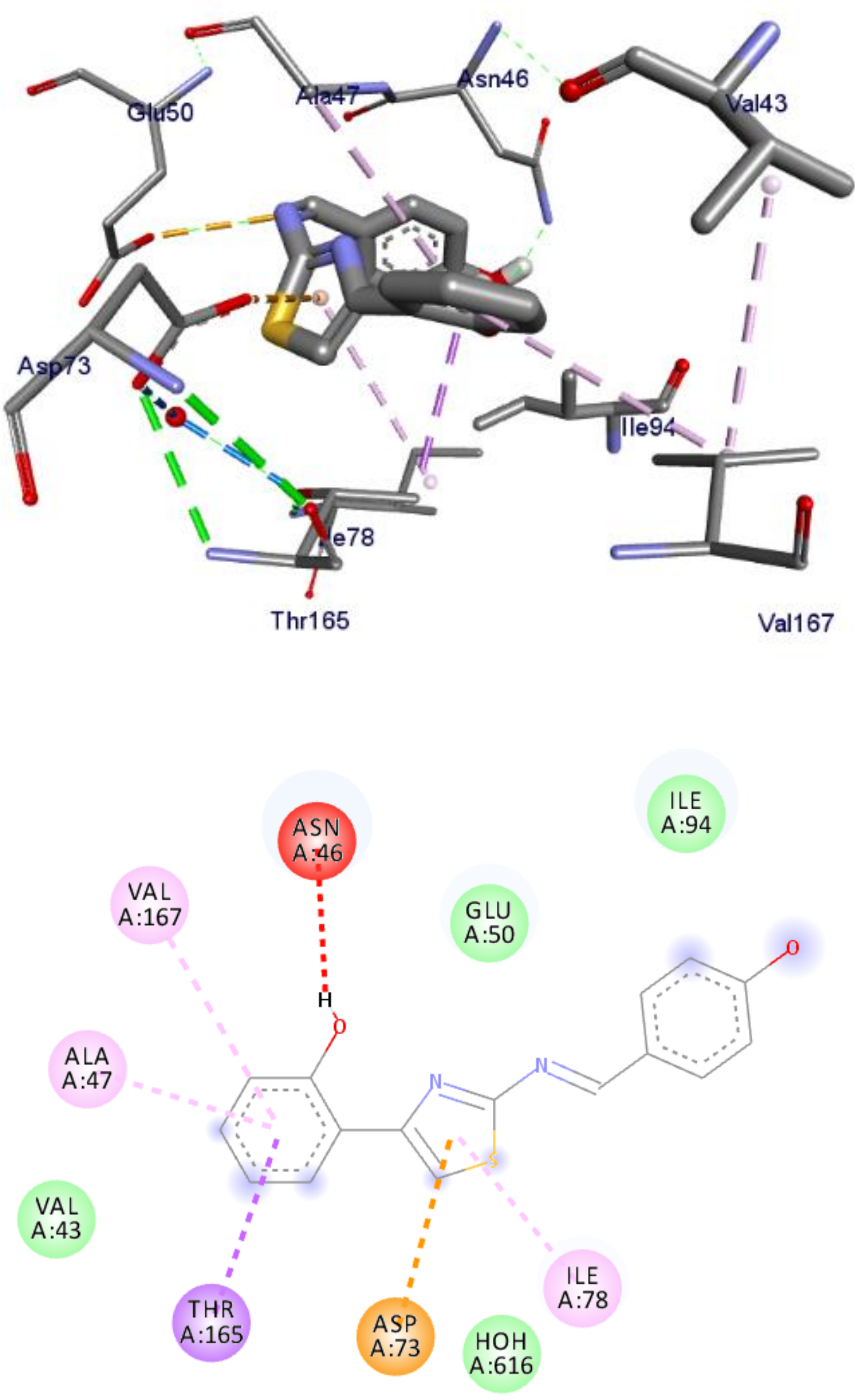

Figure S18. Possible binding interaction of compound **7** against *E. coli* DNA gyrase B (PDB ID: 6F86).

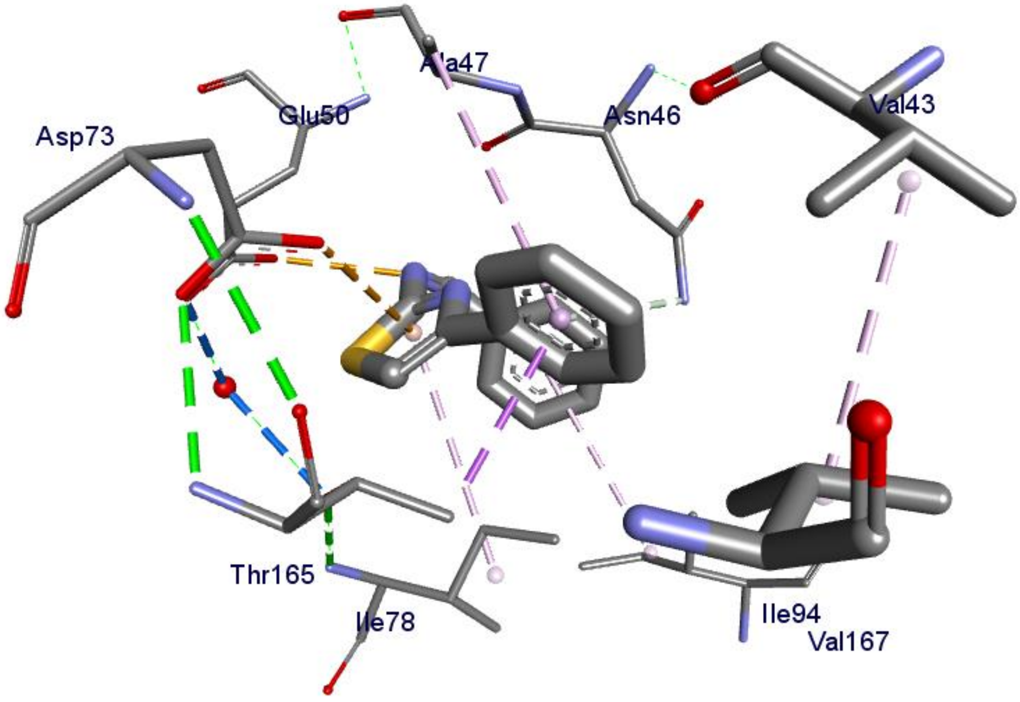
Ala47
Glu50
Asn46
Val43
Asp73
Thr165
Ile78
Ile94
Val167

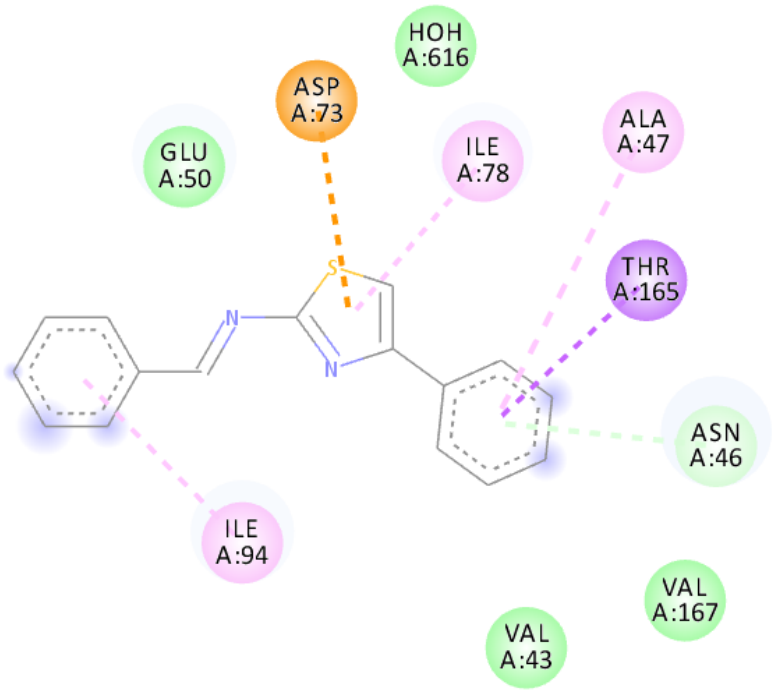

Figure S19. Possible binding interaction of compound **8** against *E. coli* DNA gyrase B (PDB ID: 6F86).

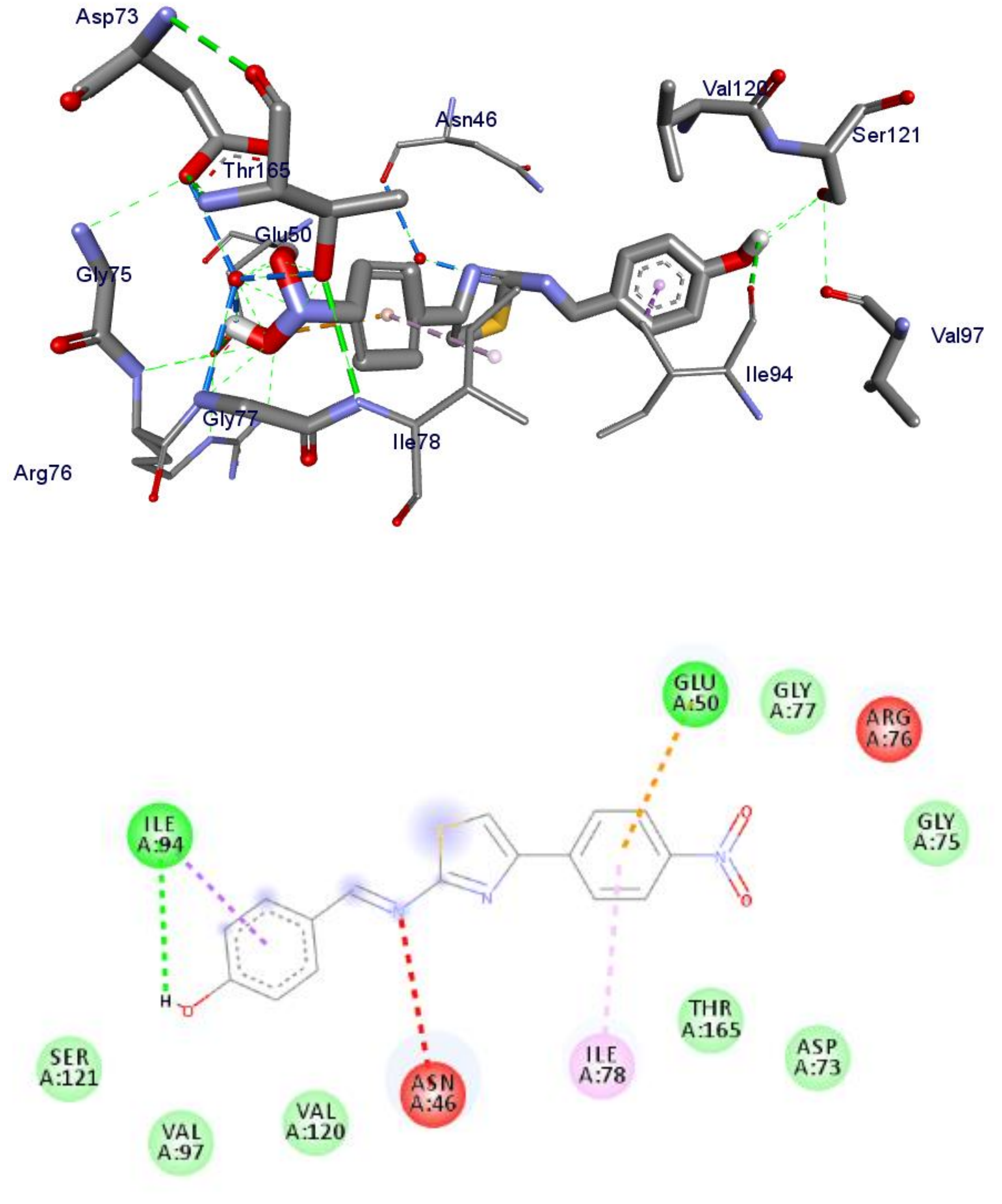

Figure S20. Possible binding interaction of compound **9** against *E. coli* DNA gyrase B (PDB ID: 6F86).

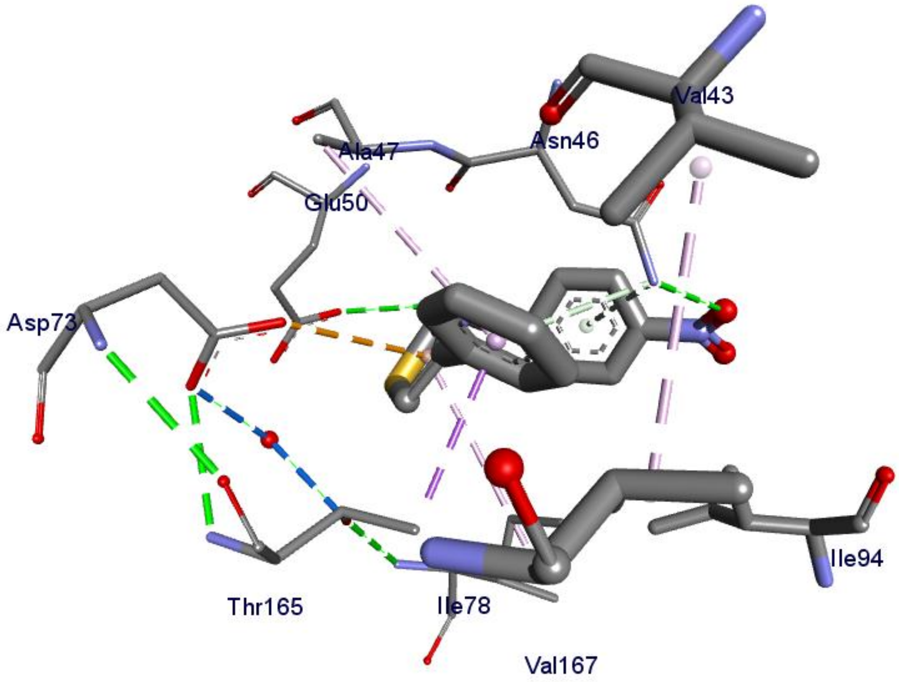

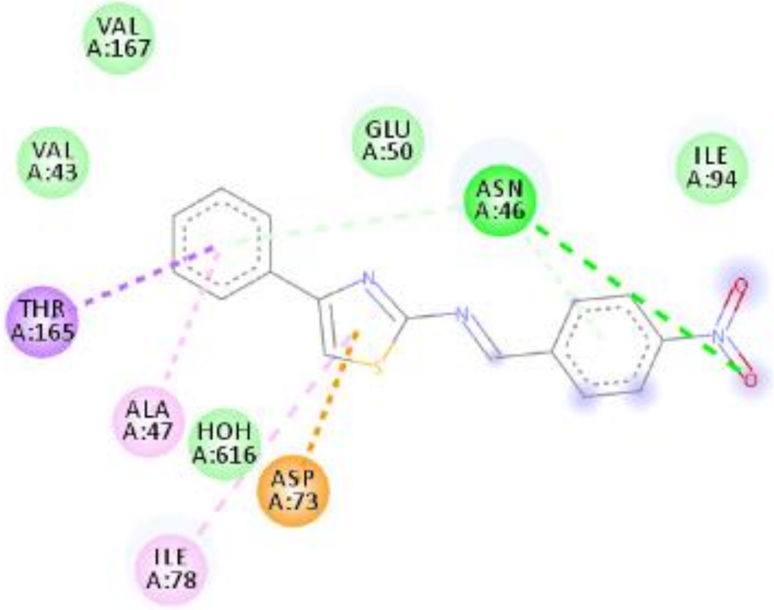

Figure S21. Possible binding interaction of compound **10** against *E. coli* DNA gyrase B (PDB ID: 6F86).

1.9 Molecular docking affinities analysis of synthesized compound (7-11) against human peroxiredoxin 5.

Table S4 Molecular docking affinities value of ligands against human peroxiredoxin 5 (PDB ID: 1HD2).

**Compounds**	**Affinity (kcal/mol)**	**H-bond**	**Residual amino acid interactions**	
			**hydrophobic/π-cation/π-anion/π-alkyl interactions**	**Van der Walls interactions**
**7**	-5.3	Cys-47, Thr-44, Gly-46	Pro-40, Pro-45, Leu-149, Arg-127	Phe-120, Gly-148, Thr-147
**8**	-5.0	Gly-46	Pro-45, Arg-127, Leu-116, Leu-149	Pro-40, Thr-147, Gly-148, Phe-120
**9**	-5.2	Cys-47, Thr-44, Arg-127, Thr-147	Pro-40	Phe-120, Ile-119, Leu-149
**10**	-5.1	Cys-47, Thr-44, Arg-127, Thr-147	Phe-120, Leu-116, Leu-149	Gly-148, Gly-41, Pro-40
**11**	-5.1	Cys-47, Gly-46, Arg-127, Thr-147	..	Thr-44, Gly-148, Leu-149
**Ascorbic Acid**	-4.9	Cys-47, Thr-44, Gly-46, Thr-147	Pro-40, Pro-45, Phe-120, Arg-127, Leu-149	--

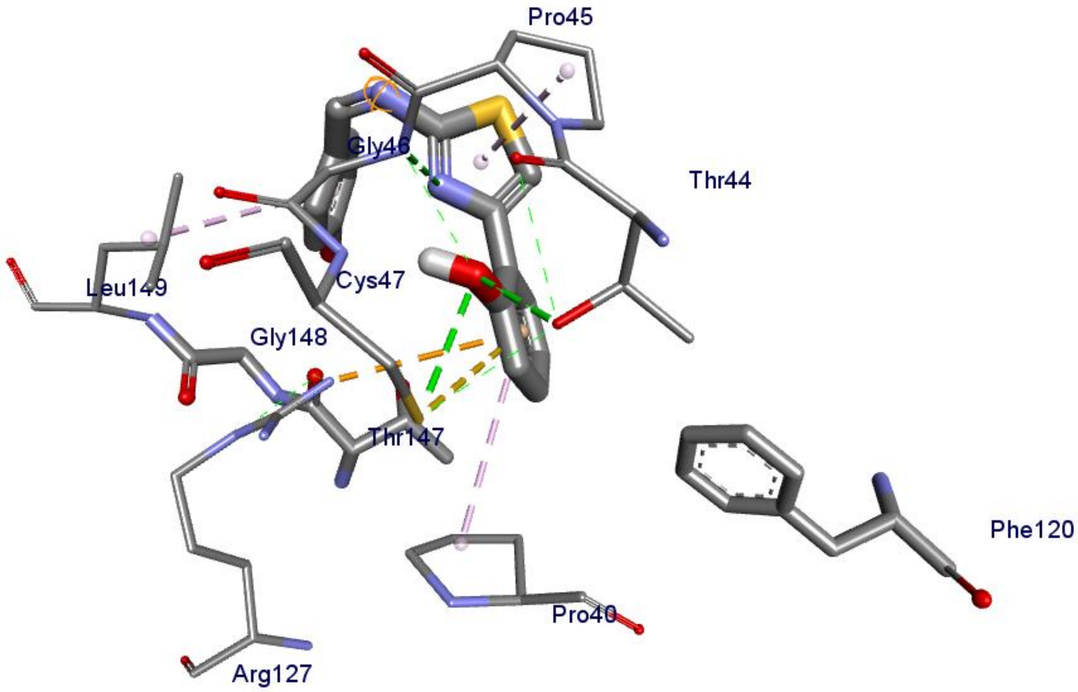

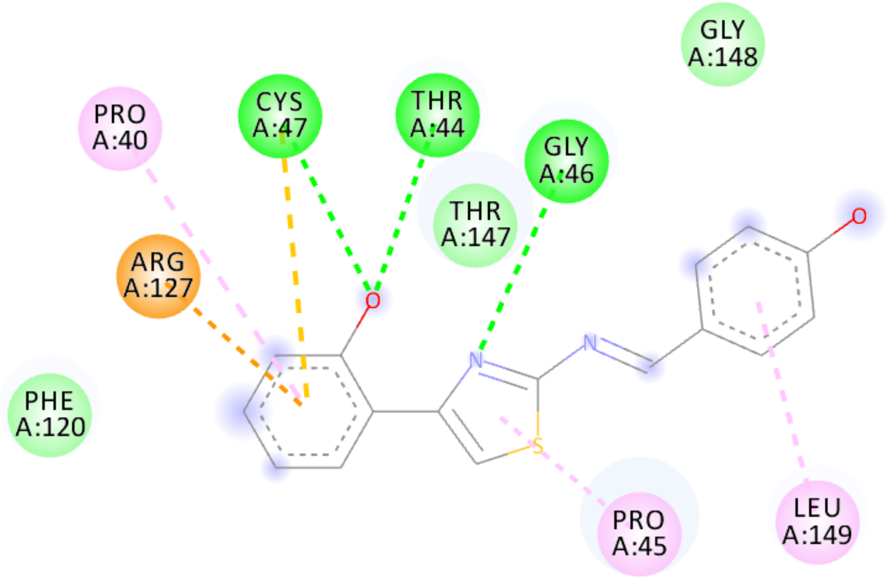

Figure S22. Possible binding interaction of compound **7** against human peroxiredoxin 5 (PDB ID: 1HD2).

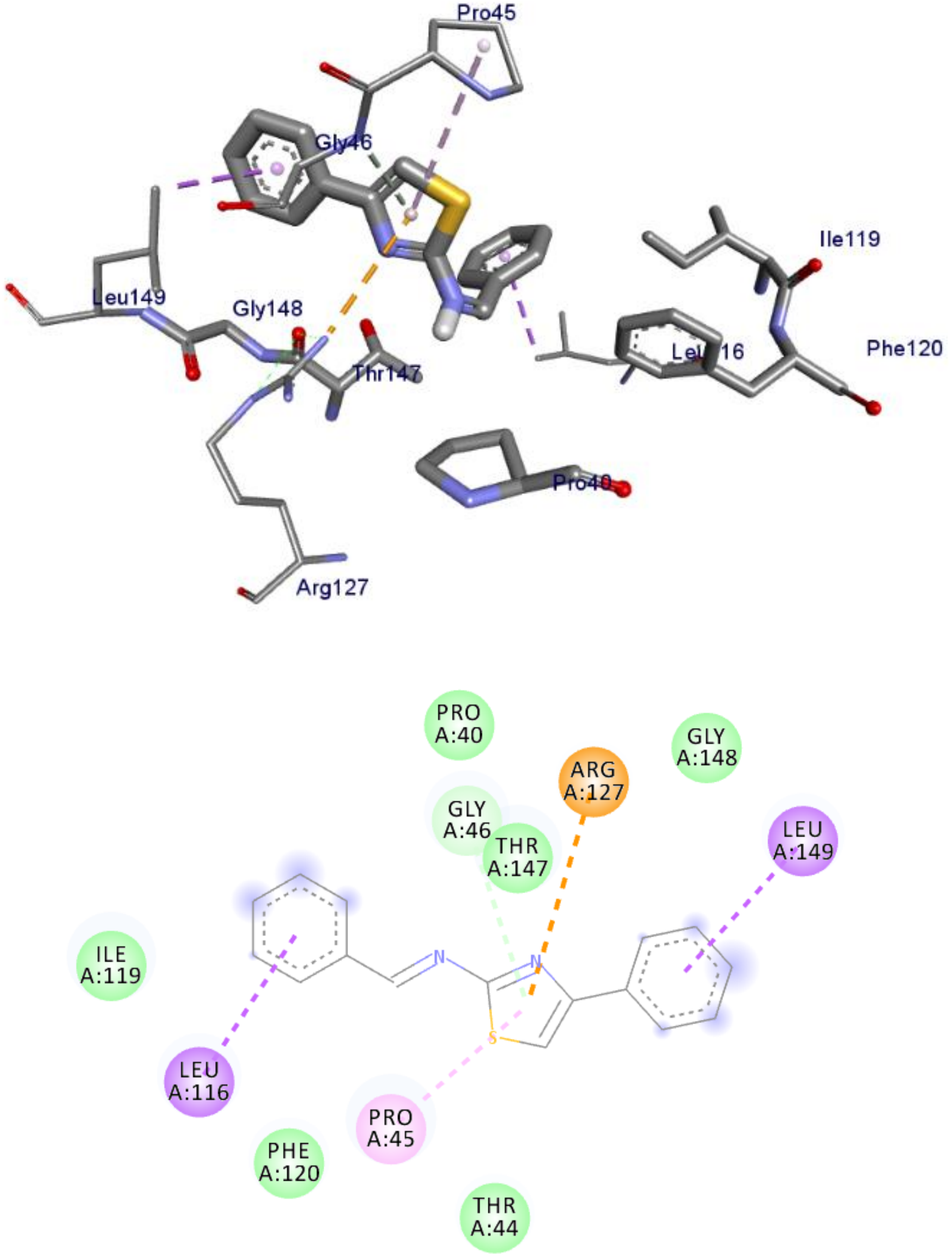

Figure S23. Possible binding interaction of compound **8** against human peroxiredoxin 5 (PDB ID: 1HD2).

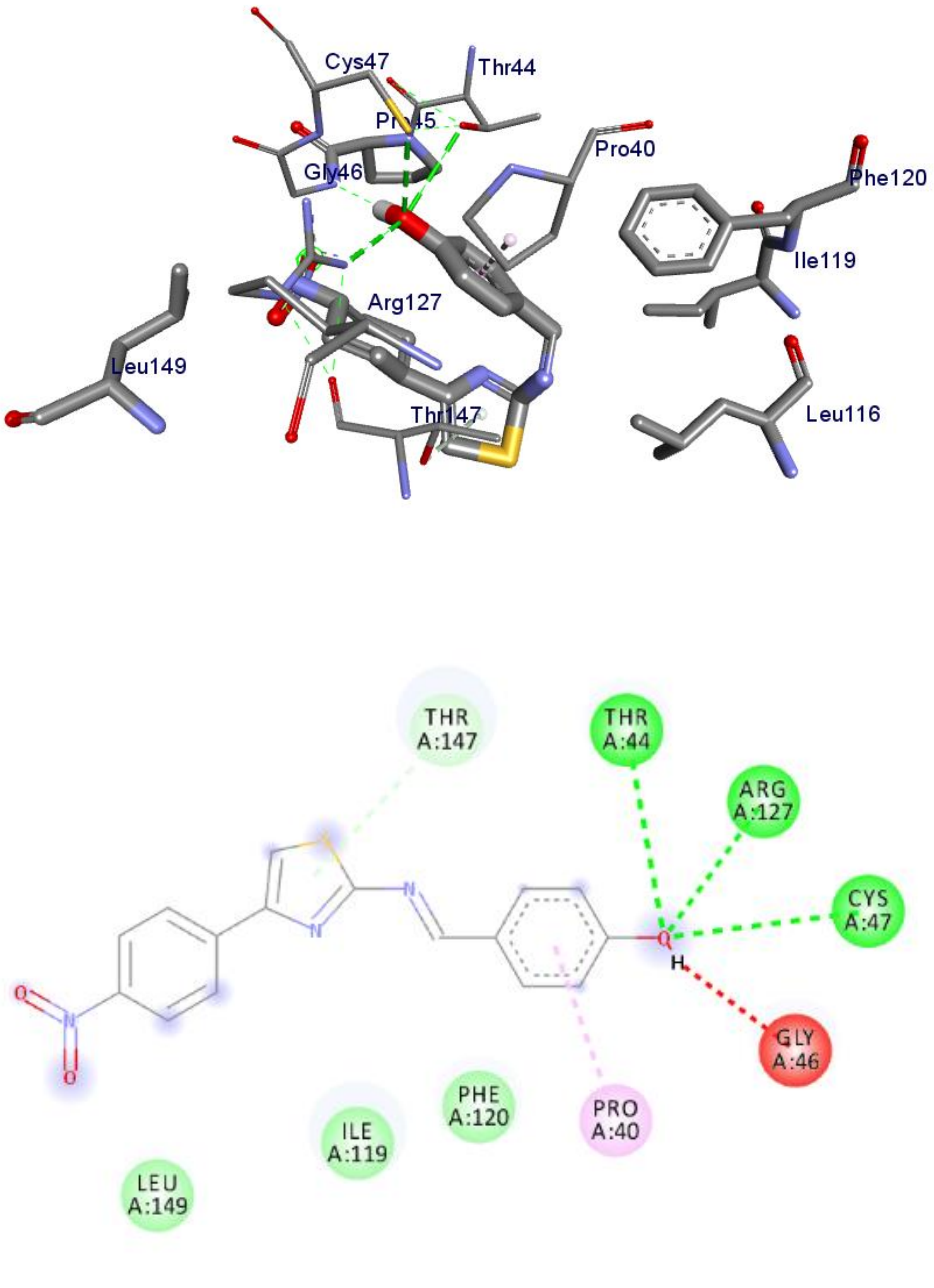

Figure S24. Possible binding interaction of compound **9** against human peroxiredoxin 5 (PDB ID: 1HD2).

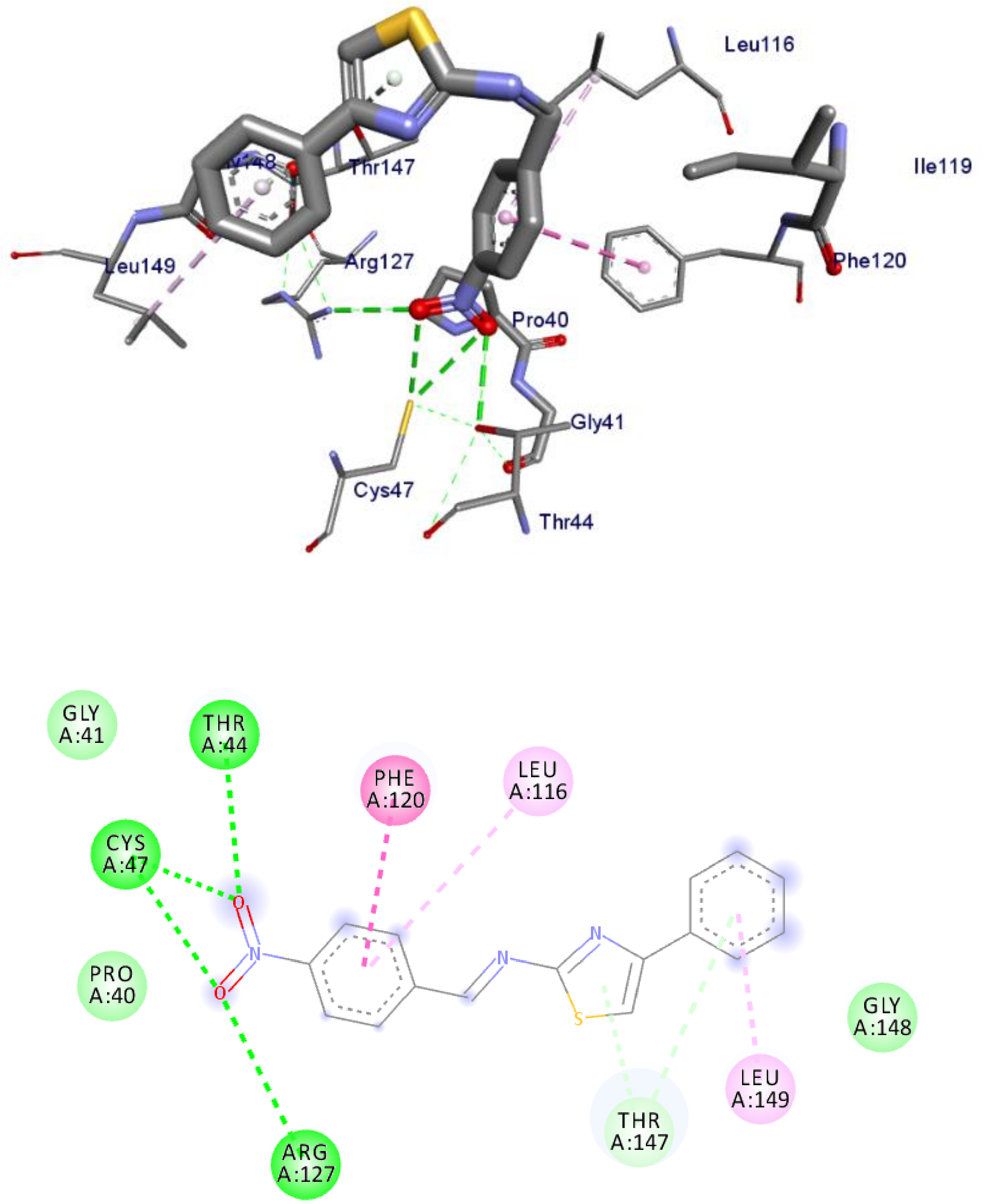

Figure S25. Possible binding interaction of compound 10 against human peroxiredoxin 5 (PDB ID: 1HD2).

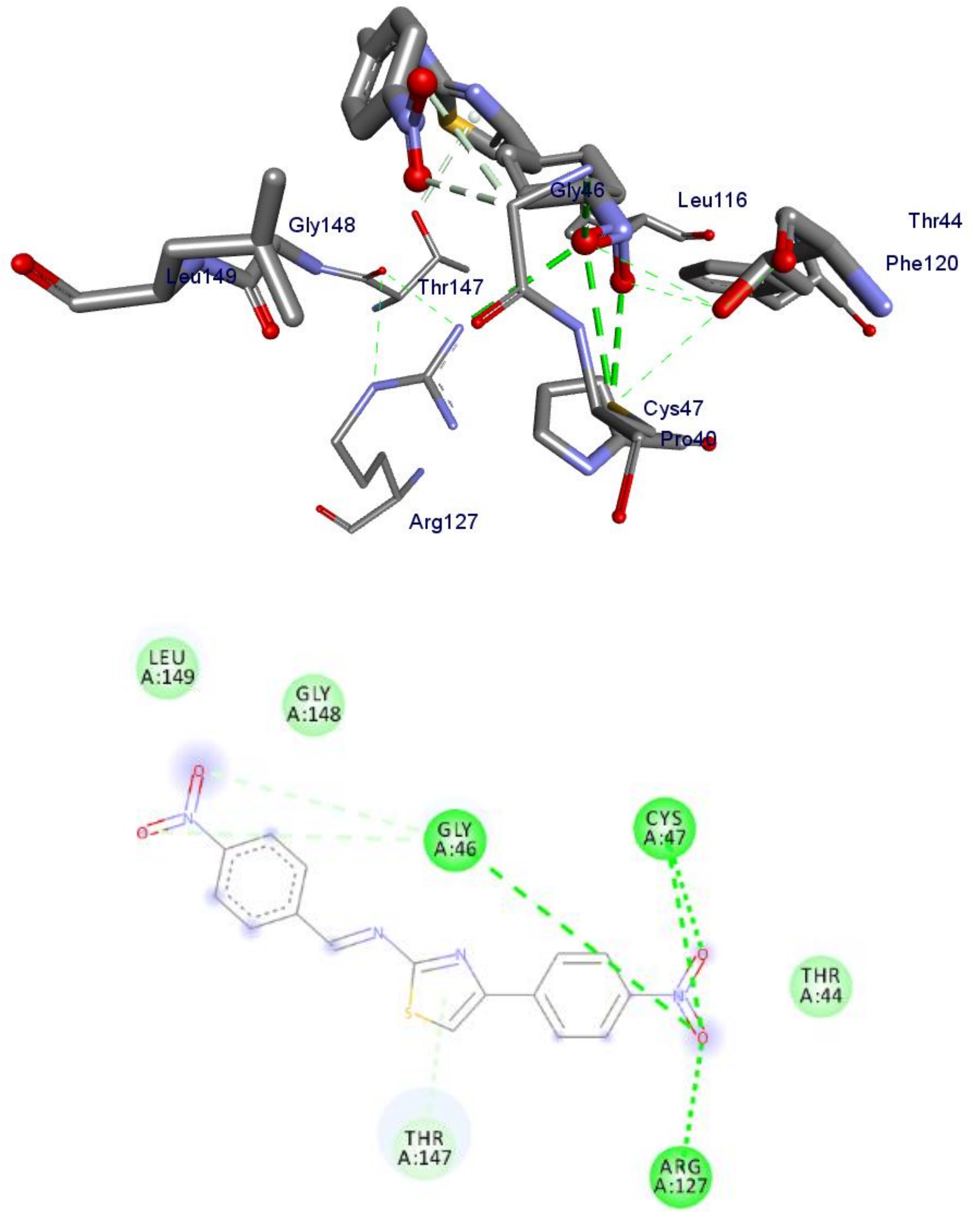

Figure S26. Possible binding interaction of compound **11** against human peroxiredoxin 5 (PDB ID: 1HD2).

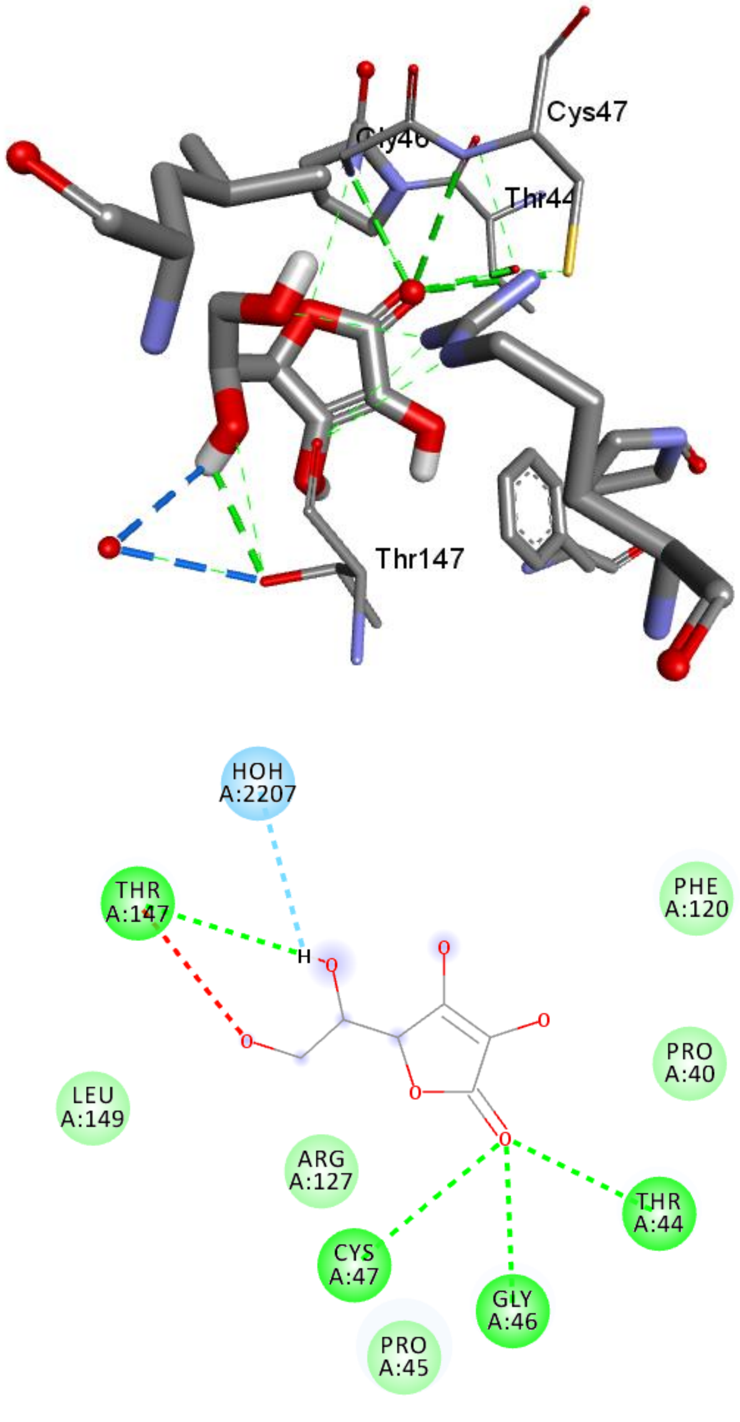

Figure S27. Possible binding interactions of ascorbic acid against human peroxiredoxin 5 (PDB ID: 1HD2).

xyz coordinates of the B3LYP-GD3/6-311++G(d,p) optimized geometries

Iodide

I	0.00000000	0.00000000	0.00000000

HI

H	-0.20354800	0.00000000	0.00000000
I	1.40354800	0.00000000	0.00000000

H_2O

O	-5.58643900	1.97747800	-0.30870800
H	-5.58643900	2.73969900	0.28113100
H	-5.58643900	1.21525600	0.28113100

Acetate ion

C	-1.83098589	0.42253520	0.00000000
O	-0.60366889	0.42253520	0.00000000
C	-2.65218202	1.72531566	0.00000000
H	-2.10329721	2.49291142	-0.50440284
H	-3.58144772	1.56117232	-0.50440284
H	-2.84237247	2.02704187	1.00880567
O	-2.59352515	-0.78718950	0.00005022

Acetic Acid

C	-1.83098589	0.42253520	0.00000000
O	-0.60366889	0.42253520	0.00000000
C	-2.65218202	1.72531566	0.00000000
H	-2.10329721	2.49291142	-0.50440284
H	-3.58144772	1.56117232	-0.50440284
H	-2.84237247	2.02704187	1.00880567
O	-2.59352515	-0.78718950	0.00005022
H	-1.99848651	-1.54053472	0.00008150

1

C	-4.73028900	1.93378000	-0.04409900
C	-3.35037900	1.86666100	0.05705700
C	-2.61320000	3.05366800	0.11029900
C	-3.26085900	4.27983200	0.06208500
C	-4.65696900	4.34559400	-0.03456300
C	-5.42129800	3.15966900	-0.08700500
H	-5.32017200	1.02692500	-0.08662000
H	-2.85004600	0.90689200	0.09636600
H	-1.53245200	3.02497000	0.18863000
H	-2.68963500	5.20175600	0.09667000
O	-5.28465700	5.55235100	-0.09631000
H	-4.63577200	6.26538500	-0.05436300
C	-6.91610700	3.09614000	-0.18166000
O	-7.46107200	2.02534700	-0.42701000
C	-7.75890500	4.32777500	0.04407800
H	-7.61431600	5.03216400	-0.77864400
H	-8.80573200	4.03129500	0.09770900
H	-7.45860800	4.85044000	0.95400600

1a

C	-4.71818000	1.92367100	-0.04259900
C	-3.33457900	1.87335300	-0.04008700
C	-2.60843000	3.06639200	0.03192300
C	-3.26993300	4.28396800	0.09837500
C	-4.66908600	4.33452700	0.08859800
C	-5.42178800	3.14268000	0.01421100
H	-5.29841700	1.01161300	-0.09725400
H	-2.82233200	0.92081300	-0.09444700
H	-1.52475000	3.04860500	0.03674600
H	-2.70858700	5.21018200	0.16103100
O	-5.31701800	5.52831400	0.16651800
H	-4.68285500	6.25446500	0.21217100
C	-6.91748200	3.05232900	-0.00619600
O	-7.46852300	1.97528100	0.13877000
C	-7.70341400	4.33453800	-0.22726400
H	-7.62810800	4.97705200	0.64703200
I	-9.83775000	4.02940000	-0.56910400
H	-7.32948500	4.88581800	-1.08569600

1b

C	-4.71525100	1.94957800	-0.05778900
C	-3.34887400	1.95485700	0.16230000
C	-2.68280300	3.17849500	0.28817100
C	-3.38664300	4.36979400	0.19281300
C	-4.76940500	4.36425000	-0.02751900
C	-5.46252600	3.14024400	-0.15588400
H	-5.24942000	1.01348400	-0.15782600
H	-2.80319600	1.02246600	0.23669500
H	-1.61309600	3.20470300	0.46063800
H	-2.87200500	5.32008300	0.28784400
O	-5.45424300	5.53568900	-0.12577700
H	-4.85611600	6.28466300	-0.01136400
C	-6.93008300	2.99180800	-0.38758700
O	-7.42480200	1.88627300	-0.55768500
C	-7.81362100	4.22662300	-0.41186900
H	-7.67301300	4.80336400	0.50242100
H	-7.49305000	4.85235400	-1.24770700
S	-9.57245300	3.72956200	-0.59032200
C	-10.45365800	5.28039800	-0.35146200
N	-11.71173000	5.14354000	-0.14899700
N	-9.73173700	6.44899500	-0.35892800
H	-8.87122700	6.50066200	-0.88229300
H	-10.26842400	7.30636200	-0.37240400
H	-12.18251800	6.04714900	-0.09076000

1c

C	-4.71215100	1.92491300	-0.06985900
C	-3.35183300	1.95994200	0.09533900
C	-2.70732100	3.20185700	0.24898700
C	-3.42438900	4.38207900	0.22750100
C	-4.81282900	4.36412500	0.05380400
C	-5.49856200	3.11575800	-0.09208200
H	-5.17290000	0.95390000	-0.20700700
H	-2.77874100	1.04275500	0.10034800
H	-1.63274200	3.23738000	0.38130500
H	-2.92131900	5.33471000	0.34310400
O	-5.52272000	5.49700800	0.02985500
H	-4.95871300	6.27327400	0.15503700
C	-6.91192200	3.02924700	-0.25488500
O	-7.53926400	1.89171900	-0.28262000
C	-7.81432200	4.20150900	-0.42800700
H	-7.73413600	4.82768700	0.46429600
H	-7.41944000	4.79648300	-1.25542700
S	-9.57214500	3.73886300	-0.70092000
C	-10.41527400	5.30284400	-0.38053700

N	-11.64159400	5.16945700	-0.05055300
N	-9.67577900	6.45519400	-0.48055400
H	-8.93302000	6.50288000	-1.16292400
H	-10.19110800	7.32145400	-0.38833300
H	-12.11474200	6.07184400	0.01042000
H	-6.97814700	1.11254700	-0.13745600

1d

C	-4.77943200	1.92755500	0.33707500
C	-3.44905200	1.80351300	-0.05033300
C	-2.76611000	2.92032900	-0.52620800
C	-3.41024500	4.15229600	-0.60850900
C	-4.74517400	4.26300300	-0.22252700
C	-5.45433100	3.15635500	0.24627100
H	-2.95390900	0.84166300	0.02469800
H	-1.72972400	2.82294800	-0.82675500
H	-2.88089800	5.02503100	-0.96988600
C	-6.89975800	3.23364300	0.73261600
C	-7.59974600	4.57602800	0.48322600
H	-7.43299200	5.26269000	1.30849200
C	-9.02949400	2.56376900	-0.11143300
N	-7.74249500	2.26066200	-0.00461400
N	-9.95580600	1.71791800	-0.52171800
H	-9.73231600	0.75593500	-0.73704900
H	-10.91174200	2.01347500	-0.65537800
O	-6.98724000	2.98278600	2.12530300
H	-6.55886300	2.13538100	2.31336400
S	-9.39942900	4.20372300	0.35637800
H	-5.22426000	5.23097200	-0.28629400
O	-5.50421200	0.87063600	0.83893300
H	-4.97195900	0.06693300	0.88763300
H	-7.29908200	5.02336900	-0.45928200
H	-7.40575800	1.30897500	-0.11059600

2

N	-4.23062100	0.08814100	0.00006100
N	-2.24607400	-1.05914300	0.00006100
H	-1.23981800	-1.07314600	-0.00015300
H	-2.73386500	-1.94324900	-0.00003600
H	-4.74492600	0.95314700	-0.00015300
H	-4.75334300	-0.77601000	-0.00003700
S	-2.03124500	1.60272300	-0.00007500
C	-2.88593500	0.12408600	0.00033300

3

C	-4.84571500	1.90030200	0.33329900
C	-3.45664300	1.84786900	0.19347700
C	-2.74995000	2.96582600	-0.24362600
C	-3.42656900	4.14726000	-0.54329300
C	-4.81268300	4.19140400	-0.41081700
C	-5.54796900	3.08014400	0.02235300
H	-2.93149500	0.93048500	0.44021600
H	-1.67238400	2.90878200	-0.34652800
H	-2.88457500	5.02055000	-0.88577000
C	-7.01679500	3.17815400	0.16324500
C	-7.64672300	4.17139100	0.85069700
H	-7.21149000	4.97838300	1.41833000
C	-9.10363000	2.53277100	-0.23999700
N	-7.85026000	2.25238000	-0.45019600
N	-10.15146900	1.83705600	-0.78717500
H	-9.91413500	0.91075900	-1.11653400
H	-11.04185800	1.88438900	-0.31355900
S	-9.38026900	3.97835700	0.74368400
H	-5.35195600	5.09865500	-0.65826400
O	-5.55708900	0.83114000	0.80337500
H	-4.95578600	0.10571600	1.01006000

4

C	-4.66064800	1.86727300	-0.08494600
C	-3.29404500	1.91926100	-0.14614800
C	-2.64061600	3.17586700	-0.13809000
C	-3.37713900	4.37578200	-0.07405800
C	-4.74500100	4.32098500	-0.01462800
C	-5.42455100	3.06755900	-0.01435200
H	-5.14000500	0.89546100	-0.09409400
H	-2.70867000	1.00902700	-0.20022700
H	-2.84558400	5.31795000	-0.07150600
C	-6.81776400	3.09874700	0.06363500
O	-7.64156000	2.09836400	0.09875900
H	-5.31991100	5.23776600	0.03547800
O	-1.31623500	3.28894200	-0.19009200
H	-0.87860100	2.42653000	-0.23095500
H	-7.33616500	4.05175800	0.10630800
H	-7.21524200	1.22551100	0.07074900

5

C	-5.34037900	2.49618300	1.65075000
C	-4.05205500	2.38700200	2.18155500
C	-2.94255200	2.77000300	1.43703000
C	-3.10875500	3.26394500	0.14268700
C	-4.38804200	3.36732700	-0.38721000
C	-5.52892000	2.99932900	0.34523700
H	-3.92731600	2.00024300	3.18782500
H	-1.95224900	2.67600300	1.86679700
H	-2.25054600	3.55330200	-0.45110400
C	-6.86119600	3.14002800	-0.26446400
C	-7.21226900	3.98228500	-1.29267600
H	-6.61053700	4.71458200	-1.80719700
C	-8.98117900	2.56576600	-0.50107400
N	-7.90439800	2.35166500	0.16768500
N	-10.19342200	1.83346100	-0.15034400
H	-10.69918400	1.53465000	-0.98966300
S	-8.86943200	3.76785700	-1.74610700
C	-9.91091100	0.51678700	0.78065600
H	-10.82942700	2.43795000	0.38173000
C	-9.14602900	-0.48906900	-0.03516200
C	-9.82706300	-1.21708600	-1.01906600
C	-7.78554700	-0.73350300	0.16783100
C	-9.16282200	-2.14932400	-1.80360700
H	-10.89056700	-1.06590000	-1.17601800
C	-7.11277000	-1.67222000	-0.60474300
H	-7.23587700	-0.19422900	0.92798200
C	-7.79861100	-2.37625900	-1.59952200
H	-9.68690600	-2.71314200	-2.56514700
H	-6.05716600	-1.85601200	-0.43784900
O	-7.19550600	-3.30263500	-2.39520900
H	-6.25950800	-3.38306400	-2.17373000
O	-9.37434700	0.94739400	1.95995600
H	-8.48241800	1.31492500	1.79488800
H	-4.51196100	3.72451700	-1.40251500
O	-6.43029800	2.12926300	2.39689400
H	-6.14254400	1.85649800	3.27690200
H	-10.93004000	0.18285100	0.97127600

6

C	-4.44356300	3.41696600	-0.83037700
C	-3.33253200	3.97400400	-1.46399400
C	-3.32314500	5.32269200	-1.80104600
C	-4.42884600	6.12445300	-1.51217600
C	-5.53526700	5.56637600	-0.88680800
C	-5.56725900	4.20954100	-0.52334700
H	-2.47799400	3.34465100	-1.68790400
H	-2.45516900	5.74309600	-2.29479400
H	-4.43341300	7.17246900	-1.78433500
C	-6.74915000	3.67233500	0.16376800
C	-7.66226700	4.35649000	0.89308800
H	-7.67193800	5.40456600	1.13989400
C	-8.18833700	1.94975500	0.74032000
N	-7.07466800	2.30987600	0.09859500
N	-8.64523400	0.69497000	0.80751000
S	-8.94861000	3.32470900	1.47753000
C	-8.17713200	-0.37799900	-0.10117300
H	-9.51654100	0.53738000	1.29446800
C	-8.87229600	-1.67140600	0.23579300
C	-9.44289600	-2.43920600	-0.78355600
C	-8.90748200	-2.15306500	1.54892500
C	-10.03742700	-3.66320400	-0.50413500
H	-9.42353500	-2.07966300	-1.80635100
C	-9.50105300	-3.37640200	1.84064700
H	-8.47504600	-1.57588500	2.35939300
C	-10.06551400	-4.13551300	0.81061800
H	-10.48220500	-4.26014700	-1.29078800
H	-9.52736200	-3.74036800	2.86205600
O	-10.66060200	-5.34319500	1.02728600
H	-10.61767700	-5.57884400	1.96205800
O	-6.75902600	-0.45597600	-0.05731000
H	-6.48270400	-0.89038500	0.76112000
H	-6.40244600	6.18477300	-0.69096300
O	-4.47944400	2.09415500	-0.47777300
H	-3.68718200	1.63219800	-0.77738500
H	-8.39934100	-0.08004200	-1.12785400
H	-6.42733200	1.61430700	-0.27363300

7

C	-1.04701100	0.46564200	-1.59367000
C	-2.31265900	1.02164700	-1.07745800
C	-2.31478100	1.69536400	0.15798900
C	-3.47050700	2.24333500	0.70219800
C	-4.67800700	2.12843300	0.01362100
C	-4.71077900	1.46706800	-1.20949400
C	-3.54753500	0.91823700	-1.75419700
H	-1.37582000	1.78118300	0.68794000
H	-3.42856300	2.75550600	1.65613800
H	-5.59006800	2.54885300	0.42112600
H	-5.64387600	1.37044700	-1.75575500
N	0.08927600	0.58636600	-0.79564200
C	-0.82870300	-0.16395400	-2.79215700
S	0.83565900	-0.63552400	-2.95269300
C	1.13236900	0.04728400	-1.34536200
N	2.41298300	0.08646600	-0.82450100
C	3.02206200	-0.92963300	-0.33001400
H	4.05837200	-0.74190600	-0.04501500
C	1.90020300	-4.98135700	0.41615000
C	2.57307600	-2.30100100	-0.07985200
C	1.22515100	-2.71237100	-0.04668500
C	3.56649000	-3.26392800	0.17168800
C	0.89207400	-4.03219000	0.20084800
H	0.42904800	-1.99602200	-0.18974900
C	3.24376800	-4.59255400	0.40569500
H	4.60971200	-2.96725400	0.17443300
H	-0.14345300	-4.34745000	0.23605700
H	4.02395200	-5.32348900	0.58661300
O	1.50713000	-6.26179500	0.64184200
H	2.27259500	-6.83457200	0.77723100
O	-3.58439400	0.26681300	-2.95737800
H	-4.48564800	0.26096700	-3.30143200
H	-1.53180300	-0.35853800	-3.58169900

8

C	-0.80799700	0.13801600	-2.29143600
C	-1.87835100	1.07676800	-1.99223500
C	-1.62833400	2.24896100	-1.24487200
C	-2.65182800	3.14746900	-0.97913400
C	-3.94651600	2.90033500	-1.44835800
C	-4.20990400	1.74306100	-2.18624100
C	-3.19108000	0.83866700	-2.45481400
H	-0.62537700	2.44013600	-0.88681900
H	-2.44581900	4.04445100	-0.40654000
H	-4.74378000	3.60387000	-1.23764000

H	-5.21293100	1.54680200	-2.54700900
N	0.44105500	0.36052900	-1.76690300
C	-1.00574500	-1.01760200	-3.13849100
S	0.49695700	-1.76006700	-3.28863000
C	1.29676800	-0.54768300	-2.15573800
N	2.60701000	-0.56458200	-1.88206400
C	3.33671800	-1.41189400	-1.25939300
H	4.41225700	-1.26014800	-1.34490300
C	2.25179500	-4.77865700	1.11252600
C	2.91975700	-2.54672300	-0.43416100
C	1.60655900	-2.71255600	0.04022100
C	3.89764700	-3.49908500	-0.09842100
C	1.27892900	-3.82247500	0.80829000
H	0.85084200	-1.96625900	-0.16537000
C	3.56186000	-4.61471400	0.66086100
H	4.91646100	-3.36532100	-0.44525300
H	0.26721700	-3.94141700	1.17707800
H	4.31848600	-5.35084800	0.90440700
H	-3.39001700	-0.06381100	-3.01775000
H	1.98973100	-5.64385300	1.71041000

9

C	-0.79731000	0.18092000	-2.32593900
C	-1.87254800	1.10903200	-2.02063500
C	-1.63445900	2.26751100	-1.24778500
C	-2.66357800	3.15794700	-0.97887200
C	-3.95143200	2.91614300	-1.47006300
C	-4.20295500	1.77238900	-2.23293000
C	-3.17886600	0.87535300	-2.50447700
H	-0.63647900	2.45474800	-0.87409700
H	-2.46794100	4.04460900	-0.38705100
H	-4.75325700	3.61340200	-1.25604100
H	-5.20105700	1.58032100	-2.60901600
N	0.44273600	0.38261200	-1.76789100
C	-0.97132500	-0.94271300	-3.21593400
S	0.53687700	-1.66663300	-3.38479700
C	1.30552200	-0.50282600	-2.18472200
N	2.61098500	-0.53642500	-1.87031200
C	3.30715200	-1.42777200	-1.27719500
H	4.38651200	-1.28486900	-1.29860900
C	2.83205000	-2.61932000	-0.55621400
C	1.51627300	-2.74438900	-0.08061900
C	3.75401500	-3.65275100	-0.32542700
C	1.11840900	-3.89345100	0.58476900
H	0.80721800	-1.93750300	-0.20217400
C	3.36442500	-4.81351200	0.32784300

H	4.77632400	-3.55256000	-0.66965500
C	2.04829100	-4.91459300	0.76853000
H	0.11119900	-3.99990000	0.96086900
H	4.06074600	-5.62196400	0.49769600
H	-3.36889800	-0.01876700	-3.08372000
N	1.62472900	-6.14383100	1.46914700
O	0.45981100	-6.22033900	1.84490400
O	2.45876600	-7.02685400	1.63888200

10

C	0.72116700	-0.80905300	4.40011200
C	-0.36326300	0.07680300	4.81999600
C	-0.41554600	1.40573500	4.36604300
C	-1.43506500	2.25281100	4.77286400
C	-2.41085600	1.76645200	5.64196200
C	-2.38624700	0.45018100	6.10843300
C	-1.36611000	-0.38671000	5.69183700
H	0.35213500	1.76867500	3.69620800
H	-1.47912500	3.27596300	4.42736300
H	-3.15755700	0.09813300	6.77852600
N	1.66168300	-0.38747100	3.45828300
C	0.96462600	-2.07619000	4.83632900
S	2.40848900	-2.77260900	4.22861900
C	2.58817400	-1.27561700	3.24511100
N	3.67185300	-1.09994300	2.42899700
C	3.89472200	-1.71975900	1.32415300
H	4.86478500	-1.50661400	0.87423900
C	1.62580500	-4.49041900	-0.98770600
C	3.06830200	-2.65455600	0.56728100
C	1.67911200	-2.81718000	0.74717900
C	3.70372900	-3.41393300	-0.43243100
C	0.96601500	-3.71836100	-0.02089700
H	1.14457800	-2.21656400	1.46945600
C	3.00068800	-4.33261900	-1.19604500
H	4.76685600	-3.28736100	-0.60509300
H	-0.10278000	-3.83658000	0.10672900
H	3.50668800	-4.91915400	-1.95471400
H	-1.34494800	-1.41343400	6.03753500
O	0.87147400	-5.36441500	-1.69921500
H	1.41423600	-5.84349500	-2.33823600
N	-3.48931900	2.65583300	6.07406200
O	-4.34795500	2.20689100	6.83304000
O	-3.49232500	3.81507600	5.66083800

11

C	0.88854300	-0.86119900	4.46787300
C	-0.13462300	0.00905000	5.04621000
C	-0.15685200	1.38148700	4.74664000
C	-1.11626700	2.21142300	5.30644500
C	-2.05949200	1.66268900	6.17410400
C	-2.06340200	0.30255800	6.48908100
C	-1.10429200	-0.51675200	5.91936700
H	0.58652800	1.79201800	4.07683100
H	-1.13774100	3.26814100	5.08076700
H	-2.80971700	-0.09642500	7.16112500
N	1.77967000	-0.37891300	3.51409000
C	1.11707600	-2.17312900	4.76869600
S	2.49632100	-2.84227100	4.01011600
C	2.66034400	-1.26973300	3.16104900
N	3.68039200	-1.02214600	2.27445200
C	3.90972700	-1.67008500	1.19638300
H	4.83041300	-1.40785900	0.67564600
C	1.65623700	-4.68934900	-0.74845500
C	3.09381500	-2.71598900	0.54490600
C	1.69188600	-2.72935900	0.61974700
C	3.75765700	-3.68750500	-0.21821200
C	0.96570600	-3.71476900	-0.03103700
H	1.16390000	-1.95978100	1.16631400
C	3.04353100	-4.69263000	-0.85639900
H	4.83762000	-3.66481200	-0.30202100
H	-0.11377300	-3.73199200	0.01094700
H	3.54468700	-5.45771000	-1.43163800
H	-1.10750900	-1.57674300	6.14382500
N	-3.07465300	2.53481100	6.76883200
O	-3.89969400	2.03399100	7.53178600
O	-3.05862500	3.73068000	6.48053300
N	0.88670300	-5.74838300	-1.43110100
O	-0.33395500	-5.74284600	-1.31039800
O	1.50597800	-6.58179000	-2.08415900

References

1. Mostafa B, Habibi-Khorassani SM, Shahraki M: An experimental investigation of substituent effects on the formation of 2, 3-dihydroquinazolin-4 (1H)-ones: a kinetic study. *Journal of Physical Organic Chemistry* 2017, 30(3):e3616.
2. More PG, Karale NN, Lawand AS, Narang N, Patil RH: Synthesis and anti-biofilm activity of thiazole Schiff bases. *Medicinal Chemistry Research* 2014, 23(2):790-799.
3. Lipkin A, Smirnov V: Synthesis of azomethines from 2-amino-4-phenylthiazole. *Chem Heterocycl* 1968, 4(3):423-423.
4. Amorim CR, Pavani TF, Lopes AF, Duque MD, Mengarda AC, Silva MP, de Moraes J, Rando DG: Schiff bases of 4-phenyl-2-aminothiazoles as hits to new antischistosomals: synthesis, in vitro, in vivo and in silico studies. *Eur J Pharm Sci* 2020, 150:105371.
5. Ali MM, Mahdi HS, Parveen A, Azam A: Optical properties of cerium oxide (CeO2) nanoparticles synthesized by hydroxide mediated method. In: *AIP Conference Proceedings: 2018*. AIP Publishing LLC: 030044.
